# Isoreticular Metal-Organic Framework-3 (IRMOF-3): From Experimental Preparation, Functionalized Modification to Practical Applications

**DOI:** 10.3390/polym16152134

**Published:** 2024-07-26

**Authors:** Haoyue Ning, Lixin Lu

**Affiliations:** 1 Department of Packaging Engineering, Jiangnan University, Wuxi 214122, China; haoyue_ning66@163.com; 2 Jiangsu Key Laboratory of Advanced Food Manufacturing Equipment & Technology, Wuxi 214122, China

**Keywords:** metal-organic frameworks, IRMOF-3, IRMOF-3 derivatives, preparation method, functionalized modification, practical application

## Abstract

Isoreticular metal-organic framework-3 (IRMOF-3), a porous coordination polymer, is an MOF material with the characteristics of a large specific surface area and adjustable pore size. Due to the existence of the active amino group (-NH_2_) on the organic ligand, IRMOF-3 has more extensive research and application potential. Herein, the main preparation methods of IRMOF-3 in existing research were compared and discussed first. Second, we classified and summarized the functionalization modification of IRMOF-3 based on different reaction mechanisms. In addition, the expanded research and progress of IRMOF-3 and their derivatives in catalysis, hydrogen storage, material adsorption and separation, carrier materials, and fluorescence detection were discussed from an application perspective. Moreover, the industrialization prospect of IRMOF-3 and the pressing problems in its practical application were analyzed and prospected. This review is expected to provide a reference for the design and application of more new nanomaterials based on IRMOF-3 to develop more advanced functional materials in industrial production and engineering applications.

## 1. Introduction

Metal-organic frameworks (MOFs), porous coordination polymers first proposed in the late 1990s and the main class in the field of porous solid materials, are synthesized by connecting metal nodes with various organic ligands [1]. Compared with traditional porous materials, MOFs exhibit many special properties such as a typically adjustable structure, larger surface area, highly ordered porous structure, and functional pore space [2]. These outstanding characteristics endow MOFs with enormous potential application value [3,4,5]. At present, there are mainly several typical series of MOF materials studied, such as UiOs [6,7,8], ZIFs [9,10,11], MILs [12,13,14], IRMOFs [15,16,17], etc.

**Figure 1:**
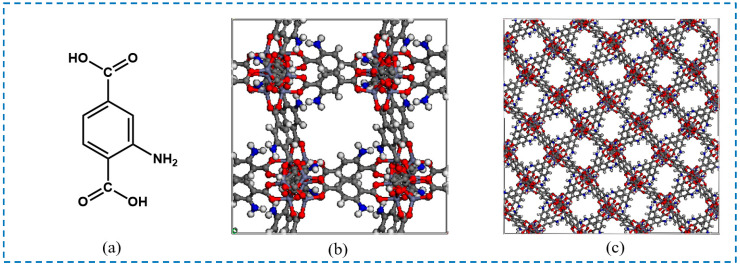
The ligand, unit cell structure, and topological structure of IRMOF-3: (**a**) ligand; (**b**)unit cell structure; (**c**) framework (the source of the IRMOF-3 CIF file database was https://bohrium.dp.tech/materials-db/database/table?type=5&utm_source=bdsem; accessed on 25 July 2022).

Isoreticular metal-organic frameworks (IRMOFs) are a series of MOF materials first discovered by Yaghi et al. in 2002 [18]. IRMOFs are a series of self-assembled MOFs with different pore diameters and similar network topologies as synthesized by the selection of different ligands [19], which are connected by Zn_4_O^6+^ tetrahedra and organic ligands and have a three-dimensional porous network structure. Different linkers of IRMOFs bring about their unique features. Among them, the isoreticular metal-organic framework-1 (IRMOF-1) is an important milestone in MOF research, but when exposed to moisture in the air, it gradually decomposes, and stability becomes the main limiting factor for its application [20]. Compared with the parent IRMOF-1, the isoreticular metal-organic framework-3 (IRMOF-3) with 2-aminoterephthalic acid (NH_2_-BDC) as the organic ligand improves its water stability [21]; exhibits more excellent performance for various applications such as adsorption, separation, catalyst, photocatalyst, etc. [22,23]; and also provides opportunities for the post-synthesis modification (PSM) of IRMOF-3. The ligand, unit cell structure, and framework of IRMOF-3 are shown in Figure 1.

The concept of PSM was first proposed by Professor Hoskins and Robson when designing three-dimensional complexes [24]. In 2007, Wang and Cohen officially defined PSM as a chemical modification after framework synthesis and successfully achieved covalent PSM of amino groups on the IRMOF-3 framework [25]. However, more strictly speaking, it only refers to modifications that form covalent bonds with the framework [26]. In recent years, the number of PSM research on IRMOF-3 with active amino sites has significantly increased. The amino group of IRMOF-3 can react with anhydride, cyano, and aldehyde groups to obtain modified products with the same topological structure but diverse functions under mild conditions [27]. In addition to modifying IRMOF-3 through PSM, there are also numerous studies utilizing the adsorption of functional substances with IRMOF-3 pore structure to functionalize it, and so on. Due to the presence of amino groups and the diversity of functionalized derivatives, IRMOF-3 has extensive applications in catalysis [28], gas storage and adsorption [29], etc.

IRMOF-3 has received widespread attention and research since its inception. This article discusses and compares the preparation methods of IRMOF-3; classifies and summarizes different types of functional modifications; and discusses its applications in catalysis, gas storage, adsorption separation, drug carriers, fluorescence detection, and other fields, including some traditional applications, as well as recent new progress. It also analyzes and prospects the urgent problems and industrialization prospects that IRMOF-3 faces in practical applications. 

## 2. Preparation Methods of IRMOF-3

IRMOF-3 is a porous material formed by the self-assembly coordination of Zn^2+^ and NH_2_-BDC, and its synthesis process is similar to the polymerization of organic compounds. At present, its preparation methods mainly include the solvothermal method, microwave-assisted method, sonochemical method, microfluidic system method, electrochemical method, heating reflux stirring method, and room-temperature stirring method.

### 2.1. Solvothermal Method

MOF materials have traditionally been prepared through the solvothermal method [30]. Usually, the reaction takes place through contact between molecules under the condition of high temperature or steam. Metal ions and organic ligands are dissolved in the corresponding solvent, heated at a high temperature for a period of time in the reactor, and then cooled to obtain MOFs [31]. 

**Figure 2:**
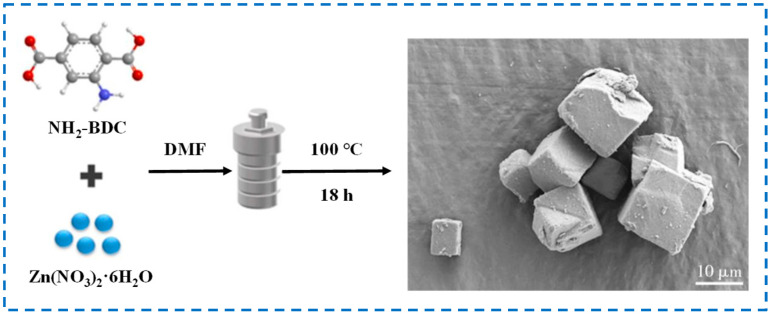
Schematic diagram and SEM diagram of IRMOF-3 prepared by the solvothermal method [35]. Copyright 2020, South China University of Technology.

The solvothermal method is the earliest and most commonly used preparation method in the related research of IRMOF-3 [32]. The main compound providing zinc source for IRMOF-3 in this method is mostly hydrated zinc nitrate. During the preparation process, hydrated zinc nitrate and NH_2_-BDC are ultrasonically dispersed in a certain volume of N-N dimethylformamide (DMF) solution, and then the mixture is transferred to a stainless-steel reactor lined with polytetrafluoroethylene. IRMOF-3 is synthesized by heating at high temperatures ranging from 100 to 130 °C for 12–24 h, followed by cooling, washing, soaking, drying, and other steps [21,33,34]. The IRMOF-3 prepared by this method has a regular structure and distinct edges (Figure 2). However, due to the long-term high-temperature heating reaction during the preparation process, the nucleation time is long, resulting in larger particles and uneven size distribution [35]. In addition, the solvothermal method often requires high temperature and pressure, with high-risk factors and high scrap rates, which has led to the gradual development of more IRMOF-3 preparation methods.

### 2.2. Microwave-Assisted Method

The microwave-assisted method refers to the solvothermal method using microwave heating. Compared with traditional solvothermal methods, the microwave-assisted method can make nucleation more uniform and greatly reduce crystallization time [36]. The principle of microwave synthesis is to convert electromagnetic energy into thermal energy to promote the occurrence of reactions. Under the action of an external alternating electromagnetic field, polar molecules in the reactant undergo polarization and alternate orientation with changes in the polarity of the external alternating electromagnetic field. Due to frequent friction and loss between so many polar molecules, electromagnetic energy is converted into heat energy, which rapidly increases the temperature of the reaction system, leading to the bonding of metal ions with organic ligands. That is to say, microwave heating does not require thermal conduction, and microwave energy can be directly and uniformly absorbed into the entire volume of the material, making it evenly and quickly heated [37].

**Figure 3:**
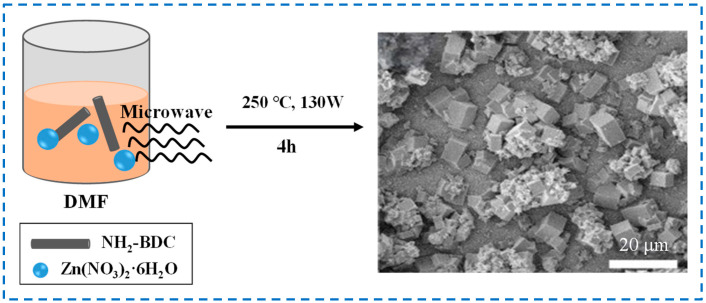
Schematic diagram and SEM diagram of IRMOF-3 prepared by the microwave-assisted method [38]. Copyright 2015, Elsevier Inc.

Figure 3 shows the schematic diagram and SEM diagram of IRMOF-3 prepared by the microwave-assisted method. A certain amount of Zn(NO_3_)_2_ ∙ 6H_2_O and 2-NH_2_-BDC mixture dissolved in a certain volume of DMF is placed in a microwave oven with an adjustable power, temperature, and time for microwave treatment to obtain IRMOF-3 products [38]. Although the particle size is relatively uniform compared with IRMOF-3 prepared by the solvothermal method, it is still larger. In addition, the microwave-assisted method has the characteristics of small reaction equipment, low energy consumption, fast reaction speed, and low production of chemical waste, but it is difficult to change the reaction conditions by adjusting the irradiation power to keep the internal conditions stable. Different instruments cannot provide exactly the same conditions, ultimately affecting the repeatability of the experiment [39]. Moreover, microwave heating requires the use of polar solvents to achieve good heating effects [40], which also has certain limitations and requirements for the choice of solvents.

### 2.3. Sonochemical Method

**Figure 4:**
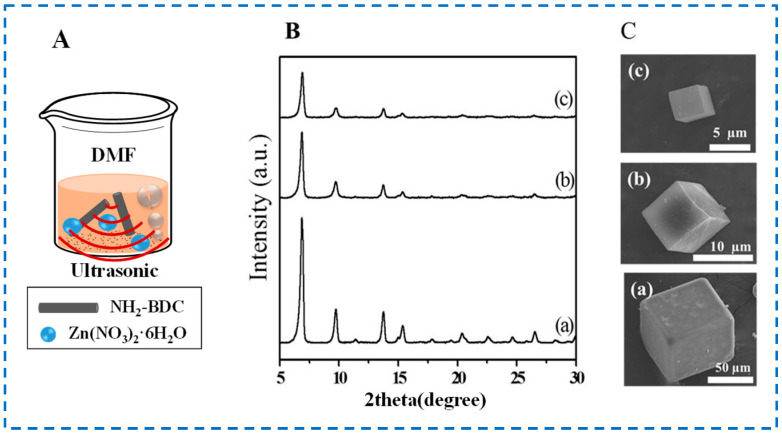
Schematic diagram (**A**) of IRMOF-3 prepared by the sonochemical method; XRD patterns (**B**), and SEM images (**C**) of IRMOF-3 samples ((a), (b) and (c) were IRMOF-3 samples synthesized by solvothermal method, microwave-assisted method, and sonochemical method, respectively.) [38]. Copyright 2015, Elsevier B.V.

The sonochemical method, also known as the ultrasonic method, uses the “cavitation” of ultrasonic to synthesize MOFs. Figure 4A shows the schematic diagram of IRMOF-3 prepared by the sonochemical method. Under the strong shock wave energy generated by ultrasound, the bubbles inside the reaction solution rapidly generate, grow, and rupture, instantly generating high temperature and pressure (>5000 K, >20 MPa), thereby promoting chemical reactions. Therefore, this method can shorten reaction time and synthesize particles with smaller particle sizes [41]. 

In Figure 4B,C, Lee et al. studied and compared the crystallinity and morphology characteristics of IRMOF-3 synthesized by the solvothermal method, microwave-assisted method, and sonochemical method, and found that IRMOF-3 synthesized by the sonochemical method had better texture characteristics and smaller particle size [38]. However, it has also been reported that MOFs prepared by the sonochemical method have various pore structures and a high probability of crystal impurity [42]. In addition, due to the presence of ultrasound, the temperature in the local area of the reaction mixture is uncontrollable, making it impossible to control the synthesis temperature. This also greatly reduces the controllability of the sonochemical synthesis of IRMOF-3.

### 2.4. Microfluidic System Method

Microfluidics technology is a scientific technology that precisely manipulates microfluidics in a microscale space. The core of microfluidic synthesis is the use of microfluidic chips. The microscale space generally consists of channels ranging from tens to hundreds of micrometers, and the volume of microfluidics is at the nanoscale, typically 10^−9^~10^−18^ L [43]. The microfluidic chip has a very high heat and mass transfer rate because the diffusion distance in the microscale reaction channel is very short, and the reactor surface area/volume ratio is several orders of magnitude higher than that of the conventional reactor [44]. In addition, in the microfluidic system, the reactant residence time is uniform, and the reaction precision and accuracy are increased, which also contributes to product control. The microfluidic system can directly control the reactant ratio, temperature, residence time, and other parameters accurately and can also realize in situ monitoring in the microreactor, thus providing possibilities for the study of reaction mechanism in the micro-environment and the design and optimization of products [45]. In general, the main characteristics of the method are uniform mixing of reactants, low reagent consumption, short molecular diffusion distance, fast reaction analysis speed, and flexible integration of multi-unit technology, etc. [46].

**Figure 5:**
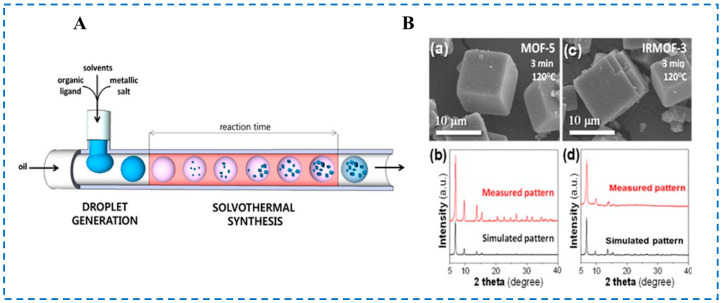
The microfluidic system (**A**), SEM micrographs (**B**-(**a**),**B**-(**c**)) and XRD patterns (**B**-(**b**),**B**-(**d**)) of MOF-5 (**B**-(**a**),**B**-(**b**)) and IRMOF-3 (**B**-(**c**),**B**-(**d**)) crystals obtained via the microfluidic approach [47]. Copyright 2013, American Chemical Society.

In the microfluidic synthesis device shown in Figure 5A, Faustini et al. used microfluidic technology to connect two T-type microchips in series, using silicone oil as a continuous phase. Metal sources and organic ligands were dissolved in corresponding solvents in the chip channel to form the microdroplet. The polytetrafluoroethylene tube was immersed in glycerol and heated at 120 °C for 3 min to rapidly synthesize MOF -5 and IRMOF-3 (Figure 5B), which was also the first time that IRMOF-3 was synthesized using this technology [47]. In the microfluidic device, the residence time of droplets during the heating phase could be controlled by controlling the flow rate of the oil phase and polar solution phase. In addition, the high surface area to volume ratio of the micro-scale space of the microfluidic device also enhanced the heat and mass transfer of the system, resulting in rapid crystallization of IRMOF-3 in the droplets. The presence of microdroplets not only increased crystallization kinetics but also avoided channel blockage due to the lack of contact between the generated IRMOF-3 particles and the channel surface. However, the particle size of the synthesized IRMOF-3 particles was still in the micrometer scale, which may have been due to the high temperature.

### 2.5. Electrochemical Method

Electrochemical synthesis of MOFs is an emerging preparation method, first reported by Mueller et al. in 2006 [48]. According to different synthesis mechanisms, electrochemical synthesis methods can be divided into two types: anodic and cathodic synthesis. The former’s reaction mechanism is the self-assembly of metal ions generated by anodic dissolution with organic linkers in solution to form MOFs; the latter is to obtain a metal source by adding metal salts (nitrate, chlorate, etc.). Some oxidizing acids (NO_3_^−^, ClO_4_^−^, etc.) form an alkaline gradient near the cathode, promote the deprotonation of organic linkers, make the organic linkers self-assemble with metal ions, and generate MOFs on the cathode surface [49]. 

**Figure 6:**
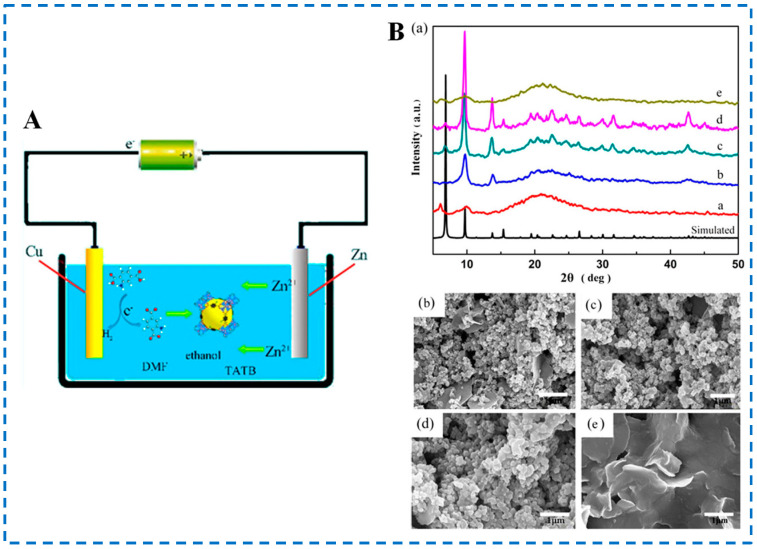
Schematic diagram (**A**), XRD patterns (**B**-(**a**)) and SEM diagram (**B**-(**b**–**e**)) of IRMOF-3 prepared by the electrochemical anode method. (**B**-(**a**)) XRD patterns of samples electrochemically synthesized at different voltages in comparison to the simulated pattern of IRMOF-3, where a–d represent the voltages at 4, 5, 6, and 7 V, respectively; (**B**-(**b**–**e**)) SEM of samples electrochemically synthesized at different voltages, where (**B**-(**b**–**e**)) represent the voltages at 4, 5, 6, and 7 V, respectively [50]. Copyright 2018, American Chemical Society.

Comparing the above two methods, the electrochemical anode synthesis system does not require the addition of metal salts, thus avoiding the interference of certain anions in the reaction. Wei et al. used this method to synthesize IRMOF-3 material for the first time [50]. As shown in Figure 6, the authors used high-purity metal zinc and copper sheets as anodes and cathodes for electrochemical reactions (electrodes need to be cleaned and pre-treated before the experiment), 2-NH_2_-BDC as an organic ligand, tetrabutylammonium bromide (TBAB) as a conductive salt, and a mixed solution of DMF and ethanol as a solvent. Under the action of an external electric field, the Zn^2+^ released by the oxidation of the anode metal zinc sheet self-assembled with the organic ligand on its surface to form IRMOF-3 (room temperature, 3 h). In addition, the influence of different voltages on the morphology of synthesized IRMOF-3 was investigated. It was found that different applied voltages could affect the release rate of metal ions, thereby affecting the ratio of metal ions to ligands to guide crystal formation. Therefore, the morphology of the material will vary depending on the applied voltage to the system. Compared with the product synthesized by the method in the above Section 2.1, Section 2.2, Section 2.3 and Section 2.4, the nanometer IRMOF-3 was synthesized by the electrochemical method. Moreover, the reaction conditions of the electrochemical method are mild, but there have been few reports on the preparation of IRMOF-3 the by this method, and a lot of research is needed in the future.

### 2.6. Heating Reflux Stirring Method

**Figure 7:**
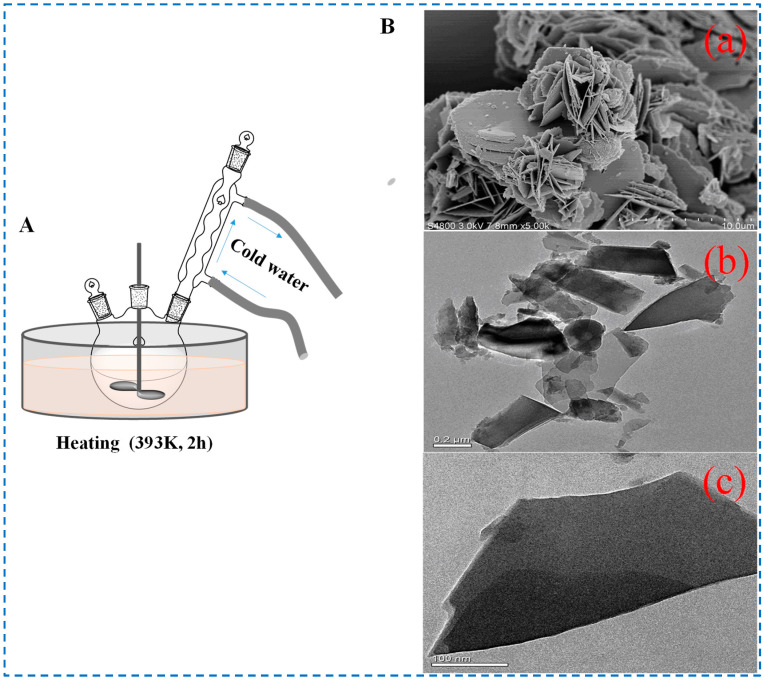
Schematic diagram (**A**), **B**-(**a**,**b**) SEM images, and (**B**-(**c**)) TEM image of IRMOF-3 prepared by the heating reflux stirring method [52]. Copyright 2018, John Wiley & Sons, Ltd.

The principle of the heating reflux stirring method is to continuously evaporate the solvent in the system through a heating reflux device, then condense it and return it to the system, which is equivalent to continuously extracting the solvent from the system while adding new solvents, guiding the formation of crystals in the material through this method [51]. The diagram of the method is shown in Figure 7. Zhu et al. prepared two solutions (Zn(NO_3_)_2_⋅6H_2_O (1 mmol) in deionized water (50 mL) and NH_2_-BDC (1 mmol) in DMF (5 mL) at room temperature, then mixed the two solutions and mechanically stirred for reflux reaction at 393 K for 2 h to prepare IRMOF-3. The SEM results in the study showed that the as-prepared IRMOF-3 sample was mostly a three-dimensional hierarchical framework constructed with a sheet-like structure, in which the sheets showed lengths ranging from 0.2 to 0.4 μm and a thickness of about 10 nm. This was also the first time that IRMOF-3 nanosheets were synthesized using the heating reflux stirring method. Different from the ordinary cube structure, the IRMOF-3 prepared by this method was a sheet structure with ultrathin characteristics, which showed good performance in the application of fluorescent probes [52]. This also provides more options and possibilities for the use of IRMOF-3 in more specific situations.

### 2.7. Room-Temperature Stirring Method

Among the above methods, the solvothermal method, microwave-assisted method, sonochemical method, microfluidic system method, and heating reflux stirring method all need to be carried out under heating conditions, and the synthesized IRMOF-3 is at the micron level due to high-temperature conditions. And the electrochemical method needs to be connected to a power supply to build a reaction path to synthesize IRMOF-3. However, the room-temperature stirring method uses hydrated zinc acetate as the metal source, and quickly pours the zinc salt into the ligand solution at room temperature by magnetic stirring to synthesize IRMOF-3. It is also known as the zinc acetate method [35], which is simple to operate, mild in reaction conditions, and saves time.

**Figure 8:**
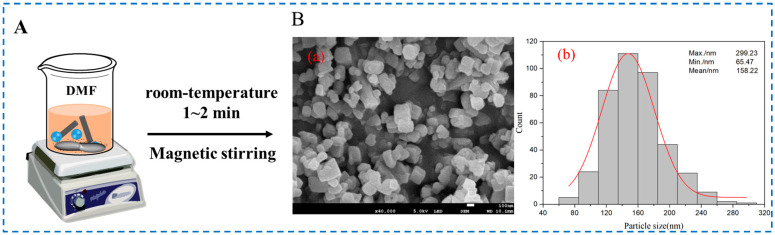
Schematic diagram (**A**), (**a**) SEM image (**B**-(**a**)), and (**B**-(**b**)) particle size statistical histogram of IRMOF-3 prepared by the room-temperature stirring method [53]. Copyright 2022, Elsevier Ltd.

Our research group used this method to prepare IRMOF-3 particles with a flat surface, with a particle size of approximately 100~200 nm (Figure 8) [53]. After dissolving zinc acetate dihydrate and 2-NH_2_-BDC in a certain volume of DMF solution and stirring the 2-NH_2_-BDC solution on a magnetic stirrer, we quickly poured the zinc acetate solution and stir for 1~2 min to obtain the IRMOF-3. Since zinc acetate can accelerate the deprotonation of 2-NH_2_-BDC and accelerate the nucleation rate, it can rapidly synthesize IRMOF-3 particles with relatively uniform particle size under simple conditions such as room temperature and stirring. However, it should be noted that in the mixing process of each experiment, the mixing speed, temperature, and time should be consistent to achieve the repeatability of the experiment and the consistency of the prepared IROMOF-3 sample.

With the gradual deepening of the research on IRMOF-3, more and more preparation methods have been deeply studied, and various methods have different advantages and disadvantages. In the future, it is still necessary to continue to explore and optimize preparation methods to achieve the convenient and efficient synthesis of IRMOF-3 for human use.

## 3. Functional Modification of IRMOF-3

In recent years, the modification of MOFs has not only become one of the hot research fields but also an important means to develop and expand the unique properties of MOFs. At present, there are many types of modification research on IRMOF-3. This section categorizes modification types based on different modification mechanisms, clarifies the reaction mechanisms of each modification type, and makes the functionalization modification research of IRMOF-3 clearer, providing a comprehensive reference for subsequent related research on IRMOF-3.

### 3.1. Covalent Post-Synthesis Modification

The covalent post-synthesis modification refers to the reaction of forming a new covalent bond between MOFs materials and the modifier. Due to the existence of the active group amino group of IRMOF-3, the covalent bond formed between the modifier and amino group to modify IRMOF-3 is the most widely used method in the functional modification of IRMOF-3 so far.

**Figure 9:**
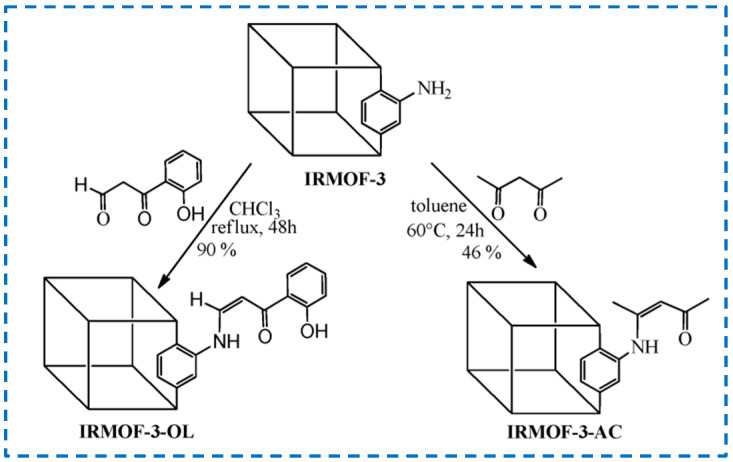
Covalent post-synthetic modification of IRMOF-3 with pentane-2,4-dione and 3-(2-hydroxyphenyl)-3-oxopropanal by the Schiff base reaction [55]. Copyright 2013, Royal Society of Chemistry.

The Schiff base reaction is a nucleophilic addition reaction between primary amine and aldehyde or ketone compounds, which is an important reaction for the formation of covalent bonds [54]. It has been reported that the amino group of IRMOF-3 reacted with pentane-2,4-dione, and 3-(2-hydroxyphenyl)-3-oxopropional, followed by the coordination of Nd^3+^/Y^3+^, which allowed the design of near-infrared light-emitting materials (Figure 9) [55]. In the reaction with pentane-2,4-dione, 46% of -NH_2_ was modified, while in the reaction with 3-(2-hydroxyphenyl)-3-oxopropional, 90% of -NH_2_ was modified, which was related to the activity of different substances in a certain reaction environment. This provides a reference for the covalent post-synthetic modification of aldehydes and ketones on IRMOF-3.

In addition to the Schiff base reaction, the amidation reaction between carboxylic acids, anhydrides, and amino groups is a common type of reaction in the covalent synthesis and modification of IRMOF-3. Liu modified IRMOF-3 with lactic acid through the condensation reaction of amino and lactic acid carboxyl groups present in the framework of IRMOF-3 [56]. The hydroxyl groups were successfully grafted onto IRMOF-3 and formed amide groups. Overall, 55% of -NH_2_ was converted into the amide, and the thermal stability of the obtained IRMOF-3-LA was the same as that of IRMOF-3. The hydroxyl and amide groups of IRMOF-3-LA were used to anchor the groups to stabilize Au^3+^ and served as catalysts. In this study, functional carboxylic acids were modified on IRMOF-3.

**Figure 10:**
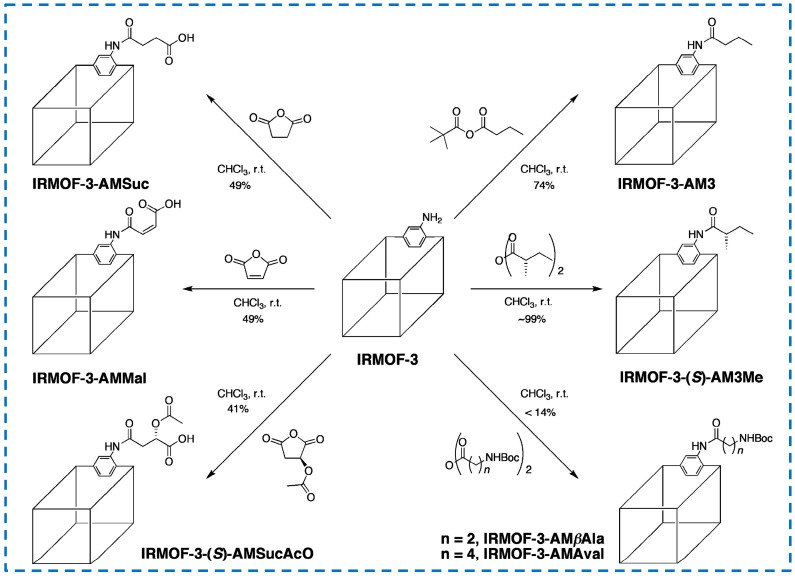
Covalent post-synthetic modification of IRMOF-3 with various acid anhydrides [58]. Copyright 2009, American Chemical Society.

Moreover, many previous studies have achieved the covalent post-synthetic modification of linear alkyl anhydrides on IRMOF-3 [25,57], while Garibay’s research has achieved the modification of different types anhydrides, resulting in new functionalized derivatives of IRMOF-3 (Figure 10) [58]. In this study, amine derivatives were introduced by modifying with N-Boc (Boc = t-Butyloxycarbonyl) amino anhydride, free carboxyl groups were generated using cyclic anhydride, chiral MOFs were generated by modifying with chiral anhydride, and IRMOF-3 was selectively converted with asymmetric anhydride containing tert butyl. In addition, multifunctional IRMOF-3 materials with up to five different substituents were successfully prepared by functionalizing a single IRMOF-3 lattice with multiple reagents. 

**Figure 11:**
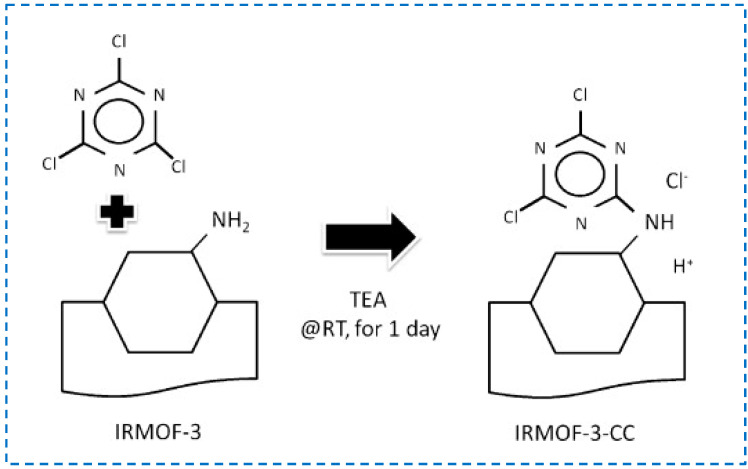
Covalent post−synthetic modification of IRMOF−3 with cyanuric chloride [66]. Copyright 2012, Elsevier B.V.

The amino group of IRMOF-3 can not only form Schiff base bonds with aldehydes or ketones, amide bonds with carboxylic acids, or anhydrides, but also condense with isocyanates to generate urea. The use of urea as an anion recognition group has been widely explored, and many studies have used MOFs containing urea for anion separation [59,60,61,62,63]. In addition, the use of urea and urea-base conjugate molecules as organic catalysts indicates that urea-modified MOFs can be used as solid organic catalytic materials [64]. IRMOF-3 has been post-synthetically modified with isocyanates to generate unprecedented, microporous urea-functionalized frameworks, and it was found that IRMOF-3 maintained good stability and high crystallinity after modification [65]. In addition, the amine group of IRMOF-3 can also react with cyanuric acid chloride to achieve the covalent post-synthesis modification. Yoo et al. modified cyanuric chloride on IRMOF-3 and formed a hierarchical pore structure through the etching process by protons released during the reaction (Figure 11) [66].

These studies on different reaction types provide powerful references for the covalent post-synthetic modification of IRMOF-3 and have also laid the foundation for further research on its covalent post-synthetic functionalization modification in the future. Here, further examples will not be repeated.

### 3.2. Tandem Post-Synthesis Modification

**Figure 12:**
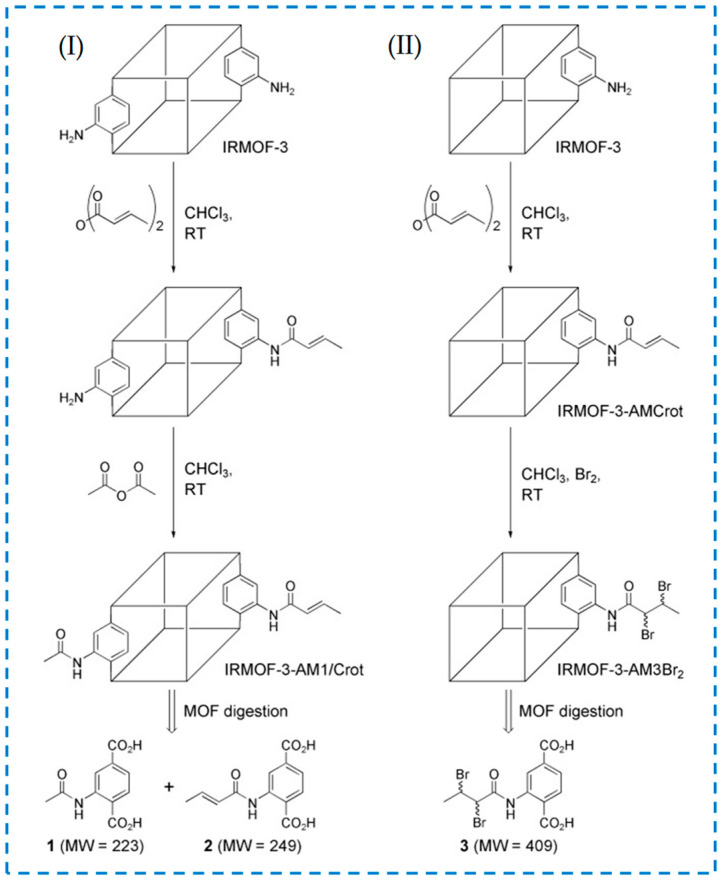
Tandem post-synthetic modification strategies of IRMOFs-3: strategy (I) and strategy (II) [67]. Copyright 2008, WILEY-VCH Verlag GmbH & Co. KGaA, Weinheim.

Most post-synthesis modification experiments of MOF materials have only involved a one-step reaction and one type of reaction. However, the stable structure of MOFs provides the possibility for them to undergo multiple continuous post-modification reactions [26]. Multiple consecutive post-modification reactions are called tandem post-synthetic modifications. Wang et al. have studied the modification after tandem synthesis and proposed two strategies for tandem modification of IRMOF-3 (Figure 12) [67]. In strategy I, one reagent was used to partially modify the active site in IRMOF-3, and then another reagent was introduced to modify the remaining active site. In strategy II, IRMOF-3 was modified with reagents containing potential functional groups, and the second step of the reaction modification was carried out based on the first step product. 

**Figure 13:**
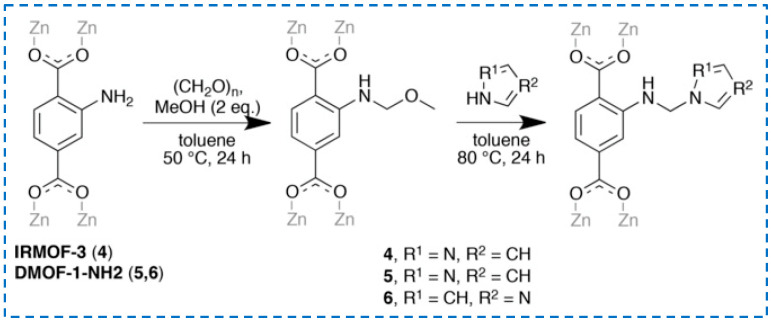
Pyrazole was modified onto IRMOF-3 through the tandem post-synthetic modification Strategy 2 [68]. Copyright 2018, Wiley-VCH Verlag GmbH & Co. KGaA.

The method of strategy II is more commonly used to prepare functionalized IRMOF-3 with excellent performance. Pyrazole was modified onto IRMOF-3 through tandem post-synthesis for selective gas adsorption or metal ion absorption (Figure 13) [68]. Based on Yoo et al.’s research [66], ammonia was added to convert the grafted cyanuric chloride into melamine [69]. Firstly, cyanuric chloride was grafted onto -NH_2_ to obtain an intermediate (IR-MOF-3-cynauric chloride) in the IR-MOF-3 group. Then, through the amination reaction, the chlorine atom of IR-MOF-3-cynauric chloride was replaced by an amine group to obtain the target product IR-MOF-3-Mel. IR-MOF-3-Mel helps to form a carbon layer with high heat resistance and graphitization level, and a relatively small number of IR-MOF-3-Mel nanoparticles can significantly reduce the fire risk of epoxy resin (EP). These studies have achieved continuous reactions on IRMOF-3 to modify it, endowing it with unique functions.

The above tandem post-synthesis functional modification is achieved by modifying IRMOF-3 through a two-step covalent post-synthesis modification in tandem. There are also studies that have achieved the functionalization of IRMOF-3 through covalent post-synthesis modification and coordination post-synthesis modification in tandem. The modified substance obtained through covalent synthesis and modification contains N and O atoms that are easy to bond with metal ions. After coordination with metal ions, it is easy to generate products with “rigid, planar, and large conjugated π bonds”, which can be used for catalysis or as luminescent materials. Liu covalently synthesized and modified IRMOF-3 with salicylaldehyde to form salicylidene imide (IRMOF-3-SI), and the integrity of the frame structure was not lost [70]. Subsequently, excessive CrCl_3_(THF)_3_ was used to treat IRMOF-3-SI to achieve chelation of Cr^3+^, which was used for the catalytic synthesis of polyethylene. In the process of processing IRMOF-3-SI with CrCl_3_ (THF)_3_, the color of the solid changed from yellow to chartreuse. In the control group experiment, the mixture of IRMOF-3 and CrCl_3_(THF)_3_ remained unchanged, indicating coordination between chromium and nitrogen atoms of the imine group, rather than a reaction with the nitrogen atoms of the amino group.

**Figure 14:**
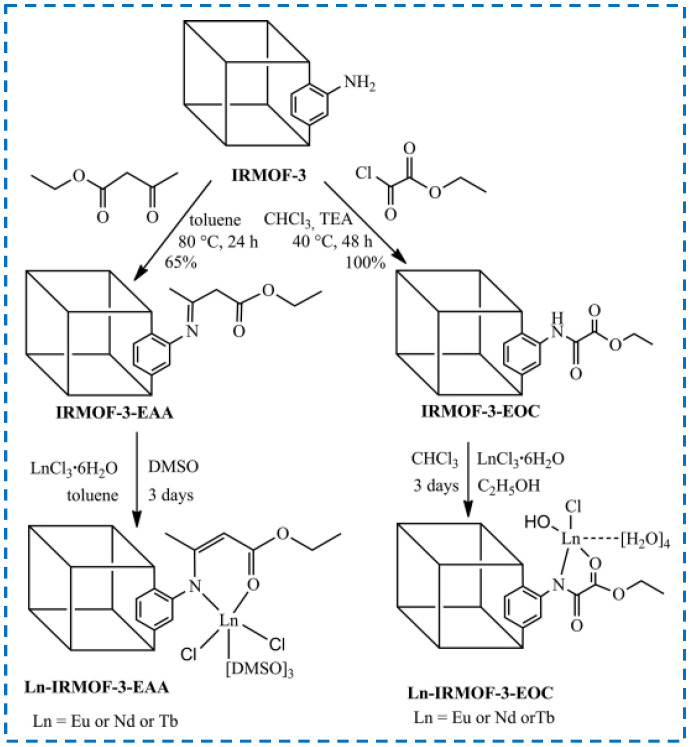
Covalent post-synthesis modification and coordination post-synthesis modification in tandem on IRMOF-3 used as the near-infrared and visible light luminescent material [73]. Copyright 2014, WILEY-VCH Verlag GmbH & Co. KGaA, Weinheim.

For the functionalization of IRMOF-3 through tandem modification of covalent synthesis and coordination synthesis, the Abdelhameed research group has conducted extensive research [55,71,72,73]. From 2013 to 2015, they covalently synthesized and modified IRMOF-3 through a series of reactions such as nucleophilic substitution, nucleophilic addition, reductive amination, and amino addition to IRMOF-3 and introduced many coordinated active sites on the MOF. Then, through the coordination of Eu^3+^, Nd^3+^, and other trivalent lanthanide ions with these active sites, various luminescent MOF materials were obtained. For example, as shown in Figure 14, IRMOF-3 was first covalently synthesized with ethyl oxalyl monochloride (EOC) and ethyl acetoacetate (EAA), and the active site was introduced to coordinate with metal ions to achieve chelation of trivalent lanthanide ions. The tandem post-synthetic modifications material was used as an effective near-infrared (Nd^3+^) and visible light (Eu^3+^ and Tb^3+^) luminescent material. In addition to using functionalized IRMOF-3 as a luminescent material, they modified IRMOF-3 with ethyl benzoyl acetate and then combined with Eu^3+^ ions to form Eu-IRMOF-3-EBA as a detection material for selective detection of organophosphorus insecticides (OPs). The complexation behavior of Eu-IRMOF-3-EBA with ethyl ion, proton and proton was studied by calculation in 2020. These studies have greatly broadened the application field of IRMOF-3.

The tandem post-synthetic modifications not only expand the scope of post-synthetic modification of IRMOF-3 but also demonstrate the feasibility of serial manipulation of IRMOF-3. The same principle can be applied to other types of reactions and more complex combinations, which add many new possibilities for developing more functional IRMOF-3.

### 3.3. Insert Reactants Synthesis Modification

In addition to the post-synthesis modification of IRMOF-3 after its synthesis, many studies have directly added functional materials as one of the reactants to the synthesis reactants of IRMOF-3, using the one-pot method to synthesize functional IRMOF-3 materials. Here, we refer to this modification method as inserting reactants synthetic modification, which mainly includes modification by the incorporation of multiple ligands, the use of various metal sources, inserting modified materials, and the formation of core–shell structures.

#### 3.3.1. Insert Mixed Ligand Synthetic Modification

**Figure 15:**
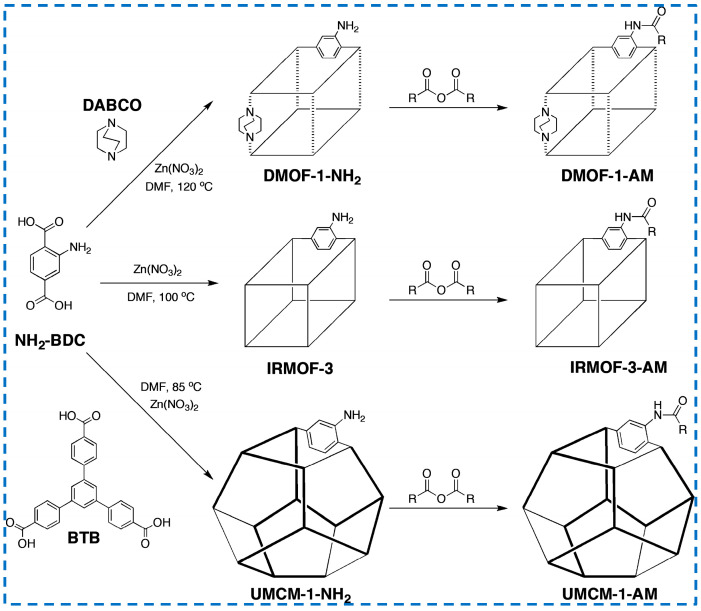
Insertion of multiple ligands in IRMOF-3 synthesis [75]. Copyright 2009, American Chemical Society.

Some studies have modified the framework structure and ligand performance of IRMOF-3 by adding different organic ligands to the synthesized IRMOF-3 reactants (Figure 15). Jie added different organic ligands of equal length (NH_2_-BDC and terephthalic acid(H_2_-BDC)) to control the density of the modified metal Ni introduced after synthesis, thereby controlling the catalytic properties [74]. In addition, studies have added triethylenediamine (DABCO) or 4,4’,4’-benzene-1,3,5-triphenyl benzoic acid (BTB) and NH_2_-BDC as organic ligand materials to prepare framework materials with non-standard structures [75]. The isoreticular framework structure of IRMOF-3 was modified, and the impact on the next modification step was studied. It was found that the modification percentage trend of the three structures was UMCM-1-NH_2_ > IRMOF-3 > DMOF-1-NH_2_. This is not only related to the different porosity of the framework structure due to different geometric lengths of ligand materials but also to the size of the free space around -NH_2_. In addition, it was found that the effect on the modification of branched anhydride seems to be more significant than that of linear anhydride, which provides an important reference for the modification of IRMOF-3.

#### 3.3.2. Insert Mixed-Metal Sources Synthetic Modification

**Figure 16:**
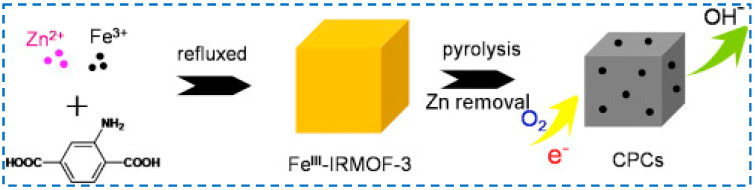
Synthesis of bimetallic organic frame material Fe^III^−IRMOF-3 by inserting Zn^2+^ and Fe^3+^ as the mixed−metal source [76]. Copyright 2016, Elsevier B.V.

In addition to modifying the structure of IRMOF-3 by introducing different organic ligands, there have also been studies on adding metal sources other than zinc to the reactants of IRMOF-3 to prepare bimetallic organic framework materials. In 2016, Wu added two metal sources (Zn^2+^ and Fe^3+^) to the synthesis of IRMOF-3 reactants to synthesize the bimetallic organic skeleton Fe^III^-IRMOF-3 [76]. After further carbonization, it was used as a catalyst for redox reactions, providing a new approach for the preparation of metal-organic skeleton derived catalysts with controllable morphology, structure, and chemical composition (Figure 16). In 2017, Ma used the same method to synthesize Fe^III^-IRMOF-3 for the detection of polluted gases [77]. Due to the unique hierarchical structure of the high specific surface area, rich exposure to active site and oxygen adsorbed on the surface, and the interface formed between ZnO and ZnFe_2_O_4_, it showed good reproducibility and selectivity for gaseous acetone.

#### 3.3.3. Insert Modified Materials Synthetic Modification

In addition to modifying the structure of IRMOF-3 by adding different ligands and metal sources to the reactants for synthesizing IRMOF-3, there have also been studies on adding functional materials in the reactants to embed into IRMOF-3. Functional materials include graphite oxide (GO), multi-walled carbon nanotubes (MWCNT-OH), magnetic particles CoFe_2_O_4_, and so on. 

**Figure 17:**
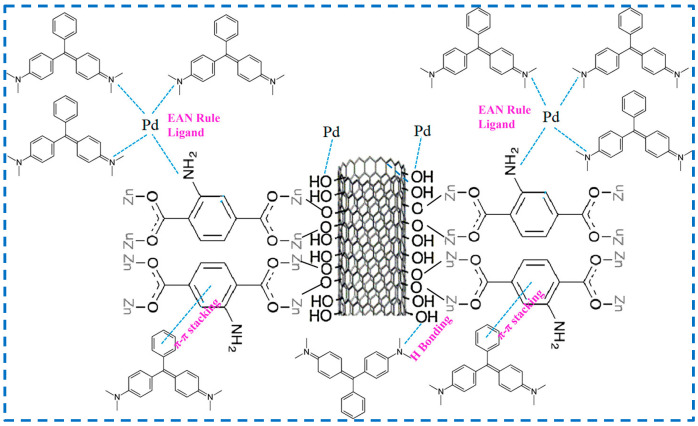
The reactants for the synthesis of IRMOF-3 inserted with MWCNT-OH [79]. Copyright 2019, American Chemical Society.

Rao added GO into the reactant of synthesized IRMOF-3 to prepare the IRMOF-3@GO complex to improve the specific surface area, dispersion force, and water stability of IRMOF-3 [78]. Then, IRMOF-3@GO was used to improve the performance of nanofiltration (NF) film adsorbed Cu^2+^ in water, which provided a new design reference for NF film to remove heavy metal ions from wastewater and demonstrated the application prospect of IRMOF-3@GO in the field of water purification. Borousan added MWCNT-OH to the reactant for synthesizing IRMOF-3, prepared a coupling material between IRMOF-3 and MWCNT-OH (IRMOF-3-MWCNT-OH), and then modified IRMOF-3-MWCNT-OH with Pd-based nanoparticles (Pd-NPs) to obtain IRMOF-3/MWCNT-OH/Pd-NPs to study their elimination of malachite green fuel (MG) (Figure 17) [79]. IRMOF-3/MWCNT-OH/Pd-NPs can greatly enhance dye removal ability, not only because there are π-π bonds between the benzene rings of MG and IRMOF-3, but also hydrogen bonds between the N of MG and the -OH of MWCNT-OH, as well as π-π bonds between the benzene ring of MG and MWCNT-OH. In addition, MWCNTs are essentially mesoporous active sites that can attach various chemicals, and Pd-NPs have the ability to bond with nitrogen atoms of three different MG molecules. The combined effect of the above reasons greatly enhances the dye-removal ability of the material. Sohrabnezhad added CoFe_2_O_4_ particles to the reactant of synthetic IRMOF-3 to embed CoFe_2_O_4_ into the structure of IRMOF-3 to prepare CoFe_2_O_4_-IRMOF-3, then introduced Zn(OH)_2_ into CoFe_2_O_4_-IRMOF-3 by impregnation method, and then converted Zn(OH)_2_ into ZnO by carbonization, realizing the incorporation of ZnO nanorods into CoFe_2_O_4_-IRMOF-3 to obtain ZnO-CoFe_2_O_4_-IRMOF-3 for the photodegradation of dyes [80]. The presence of IRMOF-3 prevents the aggregation of CoFe_2_O_4_ and ZnO nanorods to a certain extent. However, CoFe_2_O_4_ makes IRMOF-3 magnetic and can be quickly and easily separated from contaminated solutions. ZnO as a semiconductor can prevent the recombination of electron holes in IRMOF-3 during the degradation process, further enhancing dye degradation. In addition, the heterojunction between ZnO nanorods and IRMOF-3 provides a synergistic effect for the rapid transfer of photo-generated electrons to participate in photocatalytic reactions. The embedding of CoFe_2_O_4_ and ZnO in IRMOF-3 gives IRMOF-3 more functionality. These studies provide valuable references for the combination of more functional materials and IRMOF-3 so that IRMOF-3 can be given more additional functions and be more widely used.

#### 3.3.4. Core–Shell Structure Synthetic Modification

**Figure 18:**
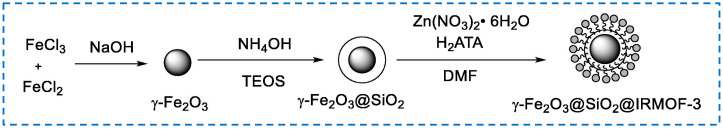
Preparation the core–shell structure of γ-Fe_2_O_3_@SiO_2_@IRMOF-3 [82]. Copyright 2020, Elsevier Inc.

Different from the synthesis modification of embedded functional materials, the core–shell structure synthesis modification is the process of forming a functionalized IRMOF-3 material with the core–shell structure using IRMOF-3 as the shell and the modified material as the core. The synthesis of core–shell structures is mostly performed directly using a one-pot method. In 2017, Li et al. synthesized uniform core–shell nanostructures (Ag@IRMOF-3) through microwave irradiation in one pot, used to catalyze the coupling reaction of three components (acetylene, aldehyde, and amine) [81]. The sizes of core (Ag) and shell (IRMOF-3) are both nanoscales and can be adjusted by controlling the crystallization time and concentration of AgNO_3_. Due to the outer layer protection of IRMOF-3, the aggregation of Ag nanoparticles can be eliminated, and Ag@IRMOF-3 can be recycled at least eight times for repeated use without the loss of activity, improving the stability of silver nanoparticles as catalysts. In 2020, Gao et al. used γ-Fe_2_O_3_ nanoparticles coated with SiO_2_ (γ-Fe_2_O_3_@SiO_2_) as the core and dispersed γ-Fe_2_O_3_@SiO_2_ into the original reactant of Zn synthesized IRMOF-3, forming the core–shell structure. γ-Fe_2_O_3_@SiO_2_@IRMOF-3 material (Figure 18) is used as a heterogeneous catalyst for cyclohexene ketone derivatives. The catalyst can be easily separated from the reaction mixture through a magnet and can be reused many times without significantly reducing the catalytic activity [82].

### 3.4. Encapsulation Post-Synthesis Modification

Different from the above post-synthesis modification, the principle of post-encapsulation synthesis modification is to functionalize IRMOF-3 by encapsulating the functional guest material with the permanent and regular pore structure of IRMOF-3. In addition, the presence of the amino group on IRMOF-3 can enhance the adsorption of IRMOF-3 to the guest material. By encapsulating different functional materials (fluorescent dyes, drugs, catalysts, etc.), IRMOF-3 materials with different functions can be obtained [83]. In conclusion, in the post-encapsulation synthesis modification, IRMOF-3 is used as the carrier material to load the guest material and functionalize it. In Section 4.4, the application of IRMOF-3 as carrier material is elaborated in detail.

Up to now, there have been many types and successful cases of the functional modification of IRMOF-3, and functional modification is the foundation for the formation of multifunctional materials. This also lays a solid foundation for the application research of IRMOF-3 in different fields.

## 4. Applications of IRMOF-3

With the increasingly mature synthesis methods of IRMOF-3 and the increasing research on functional modification, the research focus of IRMOF-3 has gradually shifted from simple material synthesis to substantive applications. IRMOF-3 and its functionalized derivatives have unique advantages in catalysis, gas storage and separation, drug carrier, fluorescence detection, etc., due to their unique structure and properties.

### 4.1. Chemical Catalysis

IRMOF-3 has unique structural characteristics that distinguish it from other catalysts, making it highly potential for application in the field of catalysis. The research and application of IRMOF-3 in the field of catalysis are also developing towards efficiency and greenness.

In 2009, Gascon et al. found that the aromatic amino groups present in the IRMOF-3 framework exhibited significant catalytic activity, with higher catalytic activity than aniline alone [84]. Based on this study, Cortese et al. believed that there was a local interaction within the IRMOF-3 framework, which could increase the alkalinity of amino groups and activate the catalyst for condensation [85]. In 2011, through density functional theory (DFT) calculations, the team found that the activity of the IRMOF-3 catalyst was independent of the alkalinity intensity. The enhancement of its catalytic activity was due to the strong interaction between the backbone center of IRMOF-3 and the by-product water molecules in the catalytic process to protect its amino groups from the influence of water particles, while the amino groups of aniline alone would form complexes with water molecules, leading to the gradual deactivation of aniline. In 2012, in order to further explore the fundamental reason for the excellent catalytic activity of IRMOF-3, Corma et al. investigated the catalytic activity of IRMOF-3 and MOF-5 with the same structure but no amino groups [86]. They found that MOF-5 also had catalytic activity, although its activity was lower than that of IRMOF-3. Through further research, it was found that IRMOF-3 could serve as the acid-base bifunctional catalyst for the Knoevenagel condensation reaction. It is believed that its high activity in the reaction comes from the synergistic catalytic effect between metal zinc ions and amino functional groups.

The above studies have gradually delved into the catalytic mechanism of IRMOF-3 based on its chemical structure. In 2017, Dai et al. studied the effect of IRMOF-3 morphology on its catalytic activity [87]. By comparing the catalytic activity of IRMOF-3 materials with three different morphologies in ethanol, it was found that IRMOF-3 with a smaller particle size exhibited the highest catalytic activity. This was attributed to the fact that smaller particle sizes facilitated the diffusion and transport of substrates in the material, thereby enhancing catalytic activity.

**Figure 19:**
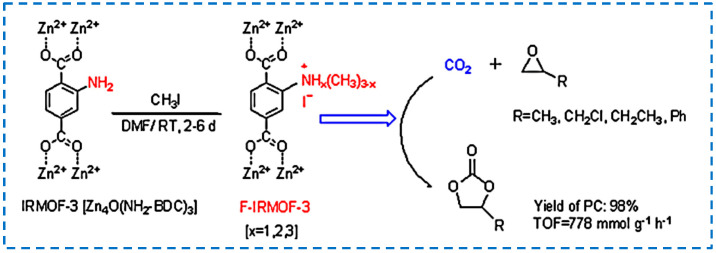
Functionalized IRMOF−3 was developed as a novel heterogeneous catalyst for the solventless synthesis of cyclic carbonates without any co−catalyst [89]. Copyright 2019, Springer Nature.

With the deepening of research on IRMOF-3 catalysis, there have been many advances in the conversion of pure IRMOF-3 into efficient catalysts in the chemical industry. The modification after synthesis can be used to develop functionalized IRMOF-3 as a catalyst. It has been studied that functionalized IRMOF-3 is obtained by modifying IRMOF-3 with methyl iodine [88]. The modified product after synthesis has excellent catalytic activity for the coupling reaction of carbon dioxide and propylene oxide, and the product yield is higher than that when IRMOF-3 and methyl iodine are used as catalysts respectively. This is because the modified IRMOF-3 with methyl iodine catalyzed through the joint action of ZnO, -NH_2_, and I^−^. Nuri et al. also reported that palladium acetate was modified on the surface of IRMOF-3 to develop a highly active and reusable heterogeneous catalyst for the Heck coupling reaction (Figure 19) [89]. In addition to modifying various functional groups or organometallic parts on IRMOF-3, Cheng et al. used IRMOF-3 as a catalyst carrier, which plays a synergistic role in catalyzing the synthesis of biodiesel [90]. In addition, research has been conducted to prepare composite-modified catalysts using magnetic Fe_3_O_4_ and CoFe_2_O_4_ with IRMOF-3. In addition to the excellent catalytic performance, the catalyst can be conveniently separated from the reaction system using an external magnetic field and can be reused [91,92,93].

The application of IRMOF-3 and its functionalized derivatives in chemical catalysis made the mechanism of action clear and made the catalysis develop towards higher catalytic efficiency, more stable catalyst, and easier recovery.

### 4.2. Hydrogen Storage

Hydrogen is known as a typical “green energy” due to its low-carbon environmental protection and high combustion value. The biggest obstacle to its widespread application is the issue of storage. MOFs have become a research hotspot in hydrogen storage applications in recent years due to their advantages such as higher specific surface area, larger pore volume, controllable structure, and modifiable pores [94]. The fact that MOF materials can be used to store hydrogen is actually due to their adsorption of hydrogen. Hydrogen storage is also a typical application of MOF materials in gas adsorption.

In 2003, Professor Yaghi’s research group at the University of Michigan in the United States first reported on the hydrogen storage performance of MOF-5, stating that there are two different hydrogen binding sites in MOF-5: one related to the Zn^2 +^ metal center and the other related to organic ligands [95]. In 2005, they prepared a series of IRMOFs materials by adjusting the organic ligands of MOF-5 and tested and analyzed their hydrogen storage performance. It was found that several IRMOFs, including IRMOF-3, had higher hydrogen storage capacity than MOF-5, which introduced IRMOF-3 into the field of hydrogen storage. Moreover, they explained the above experimental results from a structural perspective, believing that the key influence on hydrogen adsorption is the chain effect of organic ligand molecules and the changes in pore diameter and volume caused by it. Narrow and tortuous channels are more conducive to the hydrogen storage performance of MOF materials than straight channels [96].

**Figure 20:**
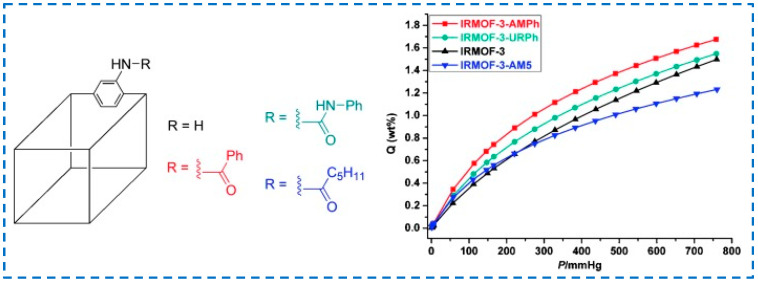
IRMOF-3 subjected to post-synthetic modification were shown to have different hydrogen adsorption properties [98]. Copyright 2010, WILEY-VCH Verlag GmbH & Co. KGaA, Weinheim.

The hydrogen storage capacity of MOF materials is not only related to their pore size but also to their interaction with H_2_. Rowsell et al. found that the interaction between MOF framework and H_2_ could be enhanced by increasing the aromaticity of organic ligands, thus improving the hydrogen storage capacity [97]. Therefore, functionalizing the ligand and introducing functional groups that can enhance the interaction between MOFs and H_2_ molecules is an effective means to improve the hydrogen storage capacity of MOF materials. In 2010, Wang et al. synthesized a series of MOFs, including IRMOF-3, and modified IRMOF-3 with benzoic acid to obtain IRMOF-3-AMPh, and its H_2_ absorption was 1.73% higher than that of unmodified IRMOF-3 (Figure 20), which was attributed to the specific interaction between H_2_ molecule and IRMOF-3 functionalized phenyl [98]. This study also demonstrated that the binding ability of H_2_ could be enhanced by adding aromatic groups to MOFs.

The research from the initial to the gradual development of IRMOF-3 in hydrogen storage applications can help researchers better understand the mechanism of its adsorption of H_2_, providing more reference for improving the design and development of IRMOF-3 in the field of hydrogen storage materials in the future.

### 4.3. Adsorption and Separation

IRMOF-3 utilized its strong adsorption capacity with guest molecules for hydrogen storage and the adsorption of single component substances, while the separation of mixed substances by IRMOF-3 was achieved based on the differences in adsorption capacity of different molecules by IRMOF-3 [99]. In addition, IRMOF-3 has inherent excellent adsorption performance and can also effectively enhance the adsorption and separation performance of IRMOF-3 by introducing specific functional groups to regulate the pore chemical properties of the material. IRMOF-3 is commonly used for the adsorption and separation of harmful substances, such as volatile organic compounds (VOCs), sulfides, and greenhouse gas CO_2_ in the atmosphere.

Volatile organic compounds (VOCs) are one of the most common air pollutants emitted by chemical, petrochemical, and related industries, including benzene series compounds, organic chlorides, freon series, organic ketones, amines, alcohols, ethers, esters, acids, and petroleum hydrocarbon compounds. Dichloromethane (DCM) and trichloromethane (TCM) are typical representatives of chlorinated volatile organic compounds (Cl-VOCs). Tian et al. studied the adsorption capacity, adsorption selectivity, and diffusion selectivity of MOF-5 and IRMOF-3 on DCM and TCM [100]. The results showed that although the porosity of IRMOF-3 decreased, the adsorption capacity for DCM and TCM increased by 5.27% and 4.5%, respectively, compared to MOF-5. The adsorption selectivity and diffusion selectivity were also higher than MOF-5. Those could all be attributed to the interaction between DCM and TCM with the amino group on IRMOF-3. Professor Yaghi’s research group, David Britt et al., studied the selective adsorption ability of six materials, including MOF-5 and IRMOF-3, on eight harmful gases (including VOCs such as gaseous tetrahydrothiophene, benzene, dichloromethane, and ethylene oxide). The results showed that the amino functionality (IRMOF-3) prove effective in adsorbing contaminants that interact strongly with this groups [101]. 

**Figure 21:**
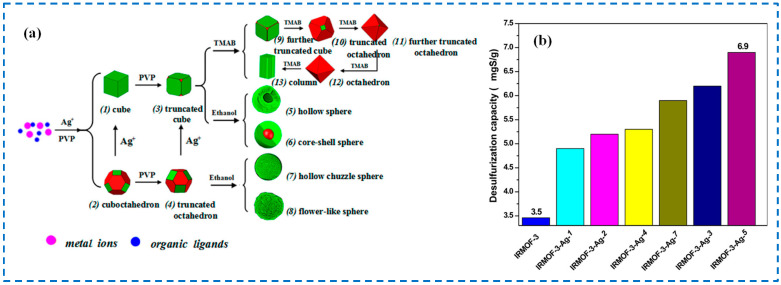
(**a**) Strategies and corresponding models for morphology-controlled IRMOF-3-Ag-n crystals by one-pot synthesis of the coordination modulation method, (**b**) sulfur adsorption capacity of a series of IRMOF-3-Ag-n crystals with different morphologies [103]. Copyright 2014, American Chemical Society.

Sulfur compounds are common impurities in fossil fuels. Low-sulfur fuels can be produced economically and efficiently by selectively adsorbing sulfides. Wang et al. studied the desulfurization performance of IRMOF-3at room temperature (dimethyl sulfur, ethanethiol, and hydrogen sulfide), with an adsorption capacity of hydrogen sulfide > ethanethiol > dimethyl sulfur, which is consistent with the interaction strength between IRMOF-3 and various sulfur compounds [102]. When adsorbing dimethyl sulfide and ethanethiol, the interaction came from the weak interaction between the amino group in IRMOF-3 and the S atom of the adsorbate, where the amino group in IRMOF-3 and the S atom of the sulfur compound played the roles of H donor and H receptor, respectively. When adsorbing hydrogen sulfide, the interaction with the S atom originated from the amino and Zn sites in IRMOF-3. The former was more like an acid-base interaction, while the latter led to the production of new products ZnS and H_2_O, severely damaging IRMOF-3. After that, Li et al. used nano silver-modified IRMOF-3 to prepare IRMOF-3-Ag-n for the adsorption of sulfide dibenzothiophene (DBT) and found that compared to the original IRMOF-3, the sulfur absorption ability of various IRMOF-3-Ag-n crystals was significantly improved (Figure 21), which may have been the result of complexation between dibenzothiophene and Ag nanoparticles [103].

Global warming poses a serious threat to the ecological environment, and CO_2_ is the main gas causing the greenhouse effect. Therefore, controlling CO_2_ emissions is of great significance. The adsorption of CO_2_ in flue gas mainly involves the separation process of CO_2_/N_2_ [104]. In 2009, Farrusseng et al. compared the heat of adsorption of seven gases (Kr, Xe, N_2_, CO_2_, CH_4_, n-C_4_H_10_, and i-C_4_H_10_) on the MOF materials, including IRMOF-3, through experiments and simulations. They found that the electric field generated by amino groups of IRMOF-3 increased the adsorption strength for more polar substances [105]. In 2010, Karra et al. investigated the influencing factors of four MOF materials, including MOF-5 and IFMOF-3, on the adsorption of CO_2_, CO, and N_2_ through atomic grand canonical Monte Carlo (GCMC) simulation and simulated the adsorption of binary mixtures of CO_2_/N_2_ and CO_2_/CO with different CO_2_ contents. The results showed that the smaller pore size of IRMOF-3 compared to MOF-5 and the presence of its amino groups could provide greater CO_2_ selectivity [106]. This indicates the inherent CO_2_ adsorption characteristics of IRMOF-3 itself. In 2016, it was reported that nitrogen-containing functional groups have CO_2_-philic properties, which can improve the selective adsorption of CO_2_ [107]. N-doped porous carbon monomer was prepared by the direct carbonization of IRMOF-3. Compared with the original IRMOF-3, it was found that its selective adsorption performance of CO_2_ was significantly enhanced because the Lewis acid-base interaction between CO_2_ and electronegative nitrogen could enhance its affinity for CO_2_. The N-doped carbon monomer obtained by IRMOF-3 carbonization was considered to be a better adsorbent for selective adsorption of CO_2_ from CO_2_/N_2_ than the original IRMOF-3. In particular, IRMOF-3/800 obtained by carbonization at 800 ℃ showed highly selective adsorption, and the adsorption capacity was larger than that of the original IRMOF-3 or even most of the MOFs. And in 2019, Ullah et al. functionalized IRMOF-3 samples using aminomethyl propanol (AMP) to obtain AMP@IRMOF-3 [108]. This study explored the potential impact of the rich surface nitrogen functional group loading on the gas adsorption performance of the IRMOF-3 skeleton. Compared with CH_4_, AMP@IRMOF-3 exhibited highly selective CO_2_ adsorption behavior, with an adsorption capacity of 2.8 times that of IRMOF-3. This indicates that the nitrogen functional group IRMOF-3 can be a promising candidate for capturing CO_2_ from natural gas. Then, in 2023, Zhang et al. conveniently prepared and purified isoreticular MOF-3 (IRMOF-3) nanosheets filled into macroporous glass fiber (GF) filters to prepare IRMOF-3 nanosheet-filled GF (IRMOF-3@GF) membranes for the first time. The IRMOF-3@GF_2.0_ membrane exhibited superior H_2_/CO_2_ separation selectivity, appropriate H_2_ permeance, and excellent durability. Additionally, GCMC simulations confirmed that the specific adsorption affinity of amino functional groups for CO_2_ over H_2_ caused a synergistic separation effect based on selective adsorption and size exclusion, leading to ultra-high H_2_/CO_2_ selectivity of the IRMOF-3@GF membrane. This innovative filled-membrane fabrication route and the presented gas separation mechanism offer a new strategy to design and prepare MOF nanosheet-based gas separation membranes [109].

In addition to its application in the adsorption and separation of harmful substances mentioned above, some studies have used IRMOF-3 and other series of IRMOFs materials coated capillary columns as gas chromatography stationary phases to achieve the adsorption and separation of persistent organic pollutants [110]. There have also been studies using IRMOF-3-coated SiO_2_/Fe_3_O_4_ magnetic particles for magnetic solid-phase extraction to determine quinolone drugs in environmental water and fish [111]. This proves the feasibility of using IRMOF-3 for chromatographic determination. In addition, IRMOF-3 was used as an efficient adsorbent for removing naphthalene from contaminated water in the study of Masoomeh et al. [112]. Emam et al. modified IRMOF-3 on cotton fabric to obtain IRMOF-3@PO@Cotton adsorbs phenol (oxygen-based compounds) and indole (nitrogen-based compounds) from fuel for fuel purification [113]. This indicates the promising application prospects of IRMOF-3 in the adsorption and separation of various substances and fields.

### 4.4. Carrier Material

Due to the special topological structure of IRMOF-3, specific substances can enter the spatial structure to the maximum extent possible. In the existing studies on IRMOF-3 as a carrier material, most of these substances have involved drug loading. Zn is one of the essential trace elements in the human body, so IRMOF-3 synthesized with Zn as the central metal is more suitable as a drug carrier. At the same time, the presence of IRMOF-3 side chain amino groups can enhance its affinity with drugs, increase drug loading, and slow down the drug release rate. In addition, its low cytotoxicity and good biocompatibility have been certified in many studies [114].

IRMOF-3, as a drug carrier, has been widely used for loading anticancer drugs, but the high toxicity of anticancer drugs can seriously harm normal cells. However, targeted research can directly target receptors or antigens specifically expressed on the surface of tumor cells, enhancing drug efficacy and reducing drug toxicity. In order to achieve targeted transportation of the anti-tumor drug 5-fluorouracil (5-FU), Yang modified IRMOF-3 with folic acid and compared its tumor targeting with the unmodified IRMOF-3-loaded 5-FU folate receptor. It was found that IRMOF-3, after being modified with folic acid, could transport 5-FU to tumor cells through the folate receptor, with a much greater targeting effect than IRMOF-3 without coupling folic acid [115]. The Li Yongji team from the Heilongjiang University of Traditional Chinese Medicine in China has achieved the characteristics of long-term, sustained release, and lung targeting of nano drug delivery systems through a series of studies on the modification of RGD peptides onto IRMOF-3 and their use for loading the anticancer drug cantharidin [116,117,118].

**Figure 22:**
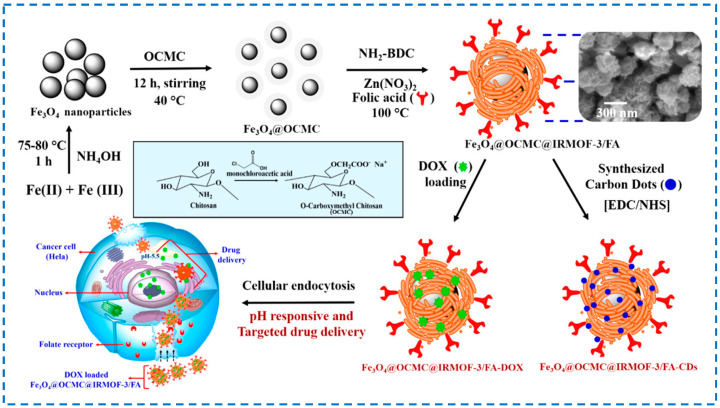
Schematic presentation of the synthetic procedure for the folic acid encapsulated magnetic nanoscale MOFs as a targeted doxorubicin (DOX) carrier [120]. Copyright 2016, American Chemical Society.

With the continuous deepening of research, the research on multifunctional and controllable release drug carriers has become a hot topic. Magnetic nanoparticles have various characteristics such as photothermal agents, magnetic resonance imaging contrast agents, and multimodal imaging [119]. Chowdhuri et al. embedded high-fluorescence carbon dots into folate-modified Fe_3_O_4_@chitosan@IRMOF-3-loaded doxorubicin nanoparticles. Chitosan could achieve the pH-controlled release of doxorubicin, folic acid could achieve targeted effects, and high-fluorescence carbon dots endowed nanoparticles with fluorescence characteristics (Figure 22) [120]. Ray et al. coupled folic acid onto core–shell porous magnetic nanoparticles Fe_3_O_4_@IRMOF-3 and studied its targeted transport of paclitaxel (FA) and magnetic resonance imaging efficacy. However, the loading capacity of FA was not high, and it was expected to modify the structure to increase the loading capacity [121].

GO has a large number of functional groups on its main chain and edge, such as epoxy (C-O-C), hydroxyl (OH), and carboxyl (COOH), which provide beneficial performance and excellent adsorption capacity. Saleheh et al. used GO to modify ZnFe_2_O_4_/IRMOF-3 magnetic nanoparticles to prepare ZnFe_2_O_4_/IRMOF-3/GO for the loading of the antibacterial drug tetracycline (TC), with a loading rate of 87% [122]. In addition, the antibacterial performance of TC was improved after being loaded, and controlled release was achieved under physiological pH environmental conditions. Mozaffari et al. used GO to modify CuFe_2_O_4_/IRMOF-3 magnetic nanoparticles to prepare GO/CuFe_2_O_4_/IRMOF-3 and then encapsulated allopurinol (Allo) in it, which can be used to improve efficacy, reduce side effects, and reduce the drug release rate [123]. It has good efficacy in reducing serum uric acid and release rate and can be a new candidate for drug delivery in maintaining liver/kidney health.

In addition to improving treatment effectiveness by increasing drug loading, it can also be achieved by reducing the ineffective release of drugs. Li et al. loaded the antineoplastic drug norcantharidin (NCTD) into IRMOF-3 and then wrapped it in a thermosensitive gel, which reduced the toxicity of NCTD and improved its bioavailability, especially avoiding its sudden release due to endocytosis or gastrointestinal absorption when be loaded only by IRMOF-3 [124]. This thermosensitive gel encapsulated IRMOF-3 has great advantages as an antineoplastic drug carrier, providing some ideas for passive targeting therapy of tumors.

**Figure 23:**
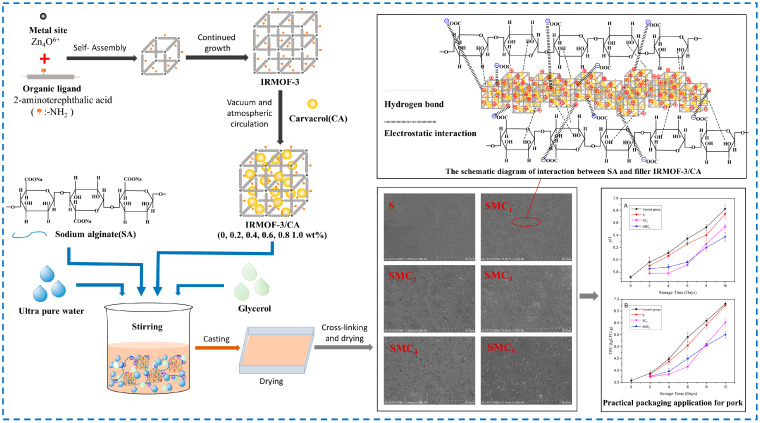
IRMOF-3 loaded CA and then added to sodium alginate matrix to prepare composite film [53]. Copyright 2022, Elsevier Ltd.

Moreover, to be used as a carrier material for drugs, IRMOF-3 is also used to load a variety of substances such as the antibacterial active substance thymol [125], dye rhodamine B, and sodium fluorescein [126], which shows that IRMOF-3 is widely used in the field of carrier materials. In addition, our research group used IRMOF-3 to load the active substance carvacrol (CA) to prepare IRMOF-3/CA assembly and added it to the sodium alginate base film to prepare the active film (Figure 23) [53]. In addition to improving the mechanical and physical properties of sodium alginate composite film, IRMOF-3 can also slow down the release rate of CA, achieving long-term antibacterial and antioxidant effects. This will introduce IRMOF-3 into the field of food active packaging research, providing a reference for its development in the food industry.

### 4.5. Fluorescence Detection

The absorbance of IRMOF-3 gave strong blue emission peaking at 450 nm upon excitation at 365 nm [126]. Its fluorescence intensity was enhanced in electron-rich systems and weakened in electron-deficient systems [127]. Therefore, based on its fluorescence enhancement or fluorescence quenching effect, it can be used for the detection of various substances such as metal ions, anions, organic small molecules, explosives, and bacteria, as well as for temperature detection and so on.

**Figure 24:**
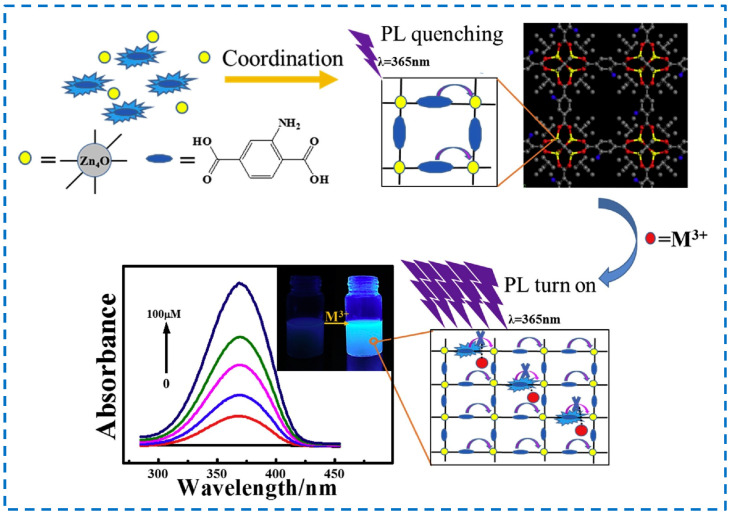
A schematic illustration of the absorbance-caused enhancement (ACE) mechanism of IRMOF-3 for the luminescence turn-on detection of M^3+^ metal ions (The lines of different colors represent the changes in absorbance changes of IRMOF-3 before and after adding metal ions.) [128]. Copyright 2018, Elsevier B.V.

Among metal ions, heavy metal ions such as Cr^6+^ and Co^3+^ pose a threat to human health and the environment, while Fe^3+^ and Al^3+^ seriously affect human physiological metabolism. Therefore, the detection of metal ions is also of great significance. Wang et al. used IRMOF-3 to detect metal ions (M^n+^) and found that when monovalent metal ions (M^+^), divalent metal ions (M^2+^), and trivalent metal ions (M^3+^) coexisted, IRMOF-3 had high selectivity for M^3+^, and the detection limits could all reach the ppm level [128]. IRMOF-3 and M^3+^ions can form a complex to absorb more energy (with higher absorbance) from the light source and then transition to the excited state. After a series of vibration relaxation processes, IRMOF-3 in the excited state begins to transfer to the ground state. During the transfer process, energy will be released, and energy release can be transferred through the form of light. During this period, The IRMOF-3/M^3+^ complex can release more energy than the original IRMOF-3 material, resulting in fluorescence enhancement (Figure 24). Therefore, IRMOF-3 can serve as a fluorescence-enhanced probe for the selective sensing and detection of M^3+^ metal ions.

In addition to the detection of M^3+^ metal ions, Wang et al. synthesized IRMOF-3-sal by modifying salicylaldehyde on IRMOF-3 and used the fluorescence enhancement effect to detect Zn^2+^ in organisms [129]. IRMOF-3-sal contained a C=N double bond, which was prone to isomerization in the excited state, so it showed very weak fluorescence. After chelating with Zn^2+^, the isomerization of the C=N double bond in its structure was blocked, and after chelating, it produced enough binding energy to form a stable excited state complex, which showed extremely strong fluorescence. However, the fluorescence intensity of IRMOF-3-sal did not change significantly after the addition of Pr^3+^ and Mg^2+^, indicating that IRMOF-3-sal has a good recognition ability for Zn^2+^ and can be used as a probe to detect Zn^2+^ in organisms.

Except for detecting cations, IRMOF-3 also has applications in the detection of anions. Wang compared the detection performance of MOF-5 and IRMOF-3 on numerous anions and found that IRMOF-3 had high selectivity for SO_3_^2−^ among many anions, while MOF-5 did not show significant detection performance for all anions [130]. This indicates that amino groups play a crucial role in the fluorescence-sensing behavior of MOF materials. This is because amino groups, as electron-donating groups, can provide more electron groups in the process of charge transfer from ligands to metal centers (LMCT). The SO_3_^2−^ can interact with amino groups to form complexes, which can hinder the LMCT effect and induce the fluorescence enhancement effect of IRMOF-3. This also indicates that IRMOF-3 has potential application prospects as a fluorescence sensor for SO_3_^2−^.

Organic small molecules are also affecting the ecological environment and human health. For example, hydroquinone (HQ) has high toxicity and low degradability, which means it will exist in the environment for a long time and cause great harm to the environment [131]. In addition, HQ can also be absorbed by the human body through the skin and respiration, causing serious damage to the human body [132]. Cao et al. used functionalized IRMOF-3 for HQ fluorescence detection for the first time, modifying Rhodamine B (RhB) onto IRMOF-3 to prepare RhB@IRMOF-3 and constructing a sensor with dual fluorescence emission. RhB is a commonly used low-toxicity fluorescent dye with red fluorescence [133]. Using the red fluorescence of RhB as a reference, the sensor was endowed with the self-calibration ability to avoid environmental interference. In addition, IRMOF-3 had blue fluorescence, which was used as an indicator. When HQ was added, it lost electrons due to oxidation and generates benzoquinone, while the unshared pair electrons on the amino group in IRMOF-3 could be transferred to benzoquinone, which weakened the blue fluorescence of IRMOF-3. Therefore, after adding HQ, the ratiometric fluorescence of the sensor decreased rapidly and reached equilibrium within 30 s, indicating its extremely fast response speed.

Aromatic explosives are serious environmental pollutants that pose a potential threat to the survival of animals and plants. In 2018, Wei et al. used IRMOF-3 to detect aromatic explosives in water (2,4,6-trinitrophenol (TNP), nitromethane (DMNB), p-nitrotoluene (4-NT), nitrobenzene (NB), and 2,4,6-trinitrotoluene (TNT)), and they found that the fluorescence intensity of IRMOF-3 was quenched to varying degrees [50]. The mechanism of fluorescence quenching of IRMOF-3 was attributed to the transfer of electrons from the excited state of the ligand to the aromatic explosives under the excitation of a certain wavelength, resulting in fluorescence quenching. In addition, had have significant differences in the fluorescence quenching effect of IRMOF-3, which may have been caused by the different electron absorption abilities of different nitro explosives. However, compared to TNP, IRMOF-3 has a lower detection limit for other aromatic nitro explosives, which is attributed to the strong intermolecular π-π stacking caused by the hydrogen bond formed between the side group primary amine group and the −OH of TNP, which is more conducive to electron transport. This indicates that TNP has a significant quenching effect on the fluorescence of IRMOF-3, implying that IRMOF-3 has the potential to detect TNP in the environment through this method. However, TNT is also a major explosive compound, but bare IRMOF-3 is less sensitive to its detection. In 2020, Devi et al. conjugated IRMOF-3 particles with NCQDs to obtain the IRMOF-3/NCQDs. Furthermore, the TNT sensing efficiency of these bare IRMOF-3 particles and IRMOF-3/NCQDs was evaluated. Sensing efficiency and selectivity of the IRMOF-3 particle for TNT elevated by increasing the electron-rich amine groups on a single MOF particle via its conjugation with NCQDs, as well as the protecting layer as formed by the NCQDs on the surface of IRMOF-3 particles. Hence, the overall quenching efficiency and the sensitivity of the IRMOF-3 particle were enhanced [134].

**Figure 25:**
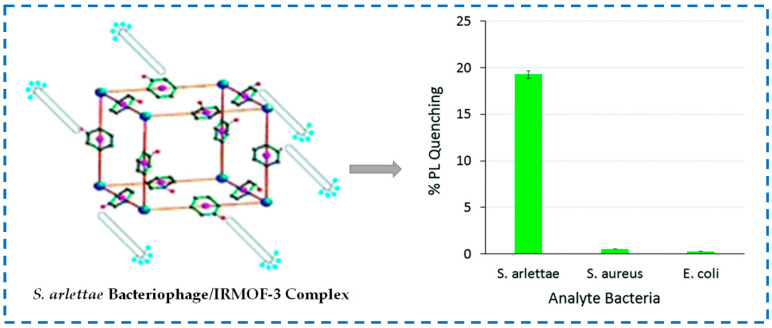
*S. arlettae* bacteriophage/IRMOF-3 complex and the specificity of detection of *S. arlettae* with the bacteriophage/IRMOF-3 complex. The sensor showed a quenching in its PL intensity only in the presence of *S. arlettae* [135]. Copyright 2016, Royal Society of Chemistry.

Moreover, IRMOF-3 can also be used for the detection of pathogenic bacteria. Bhardwaj et al. prepared the phage/IRMOF-3 by covalently attaching the phage of *Staphylococcus arlettae* (*S. arlettae*) to IRMOF-3 and used the fluorescence quenching specificity of IRMOF-3 to detect *S. arlettae* (Figure 25) [135]. Due to the specific biological recognition function of bacteriophages, phage/IRMOF-3 fluorescent biosensors had high selectivity towards *S. arlettae*. When phage/IRMOF-3 bound to *S. arlettae*, its surface was enveloped by micrometer-sized bacteria, which limited the excitation energy of IRMOF-3 and led to a loss of fluorescence intensity. Based on this study, the application of specific phage/IRMOF-3 biosensors can also be extended to several other types of pathogenic or nonpathogenic bacteria.

**Figure 26:**
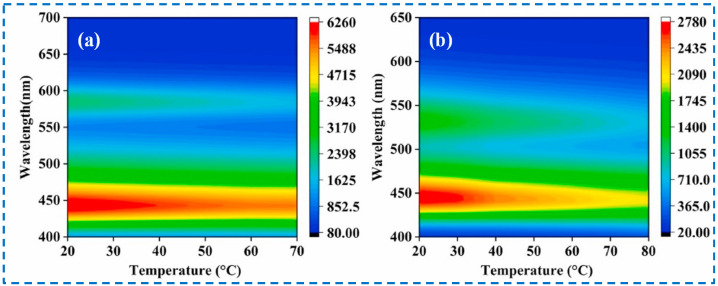
The luminescence intensity-temperature relationship based on the experimental data: (**a**) corresponds to RhB@IRMOF-3 and (**b**) corresponds to FL@IRMOF-3 [126]. Copyright 2022, Elsevier Ltd.

Surprisingly, IRMOF-3 can be used not only to detect various substances but can also to detect temperature. Traditional contact thermometers are not suitable for the temperature measurement of rapidly moving objects; therefore, non-contact temperature measurement technology based on luminescence has been increasingly studied [136]. He et al. obtained RhB@IRMOF-3 and FL@IRMOF-3 by loading two fluorescent dyes, Rhodamine B (RhB) and Fluorescein (FL), within the IRMOF-3 framework [126]. Within a certain temperature range, there is a specific relationship between the luminescence intensity and temperature of IRMOF-3, RhB, and FL, respectively. RhB@IRMOF-3 and FL@IRMOF-3 could exhibit the dual fluorescence emission characteristics of IRMOF-3 and dyes, making them excellent proportional-based luminescent temperature measurement materials. The ratio detection uses two different wavelengths of emission intensity for self-calibration, thereby eliminating interference caused by factors such as instrument efficiency, environmental conditions, and probe concentration. Additionally, it successfully overcomes the quenching effect caused by dye aggregation. According to the relationship between the luminescence intensity and temperature of RhB@IRMOF-3 and FL@IRMOF-3 (Figure 26), the corresponding actual temperature can be obtained through fluorescence intensity, making it suitable for use as a non-contact colorimetric thermometer, indicating the potential of IRMOF-3 in non-contact temperature detection. 

As more and more possibilities of IRMOF-3 and its functionalized derivatives have been explored, they have been studied and applied in many fields, and these studies have provided valuable reference ideas and laid the solid foundation for more extensive application research of IRMOF-3 and its derivatives in the future. In addition, in the application research of IRMOF-3 and its derivatives, the derivatives showed relatively excellent properties, which would stimulate more research and exploration of functional derivatives of IRMOF-3.

## 5. Conclusions and Outlook

IRMOF-3 has attracted much attention due to its large specific surface area and porosity, surface modifiability, non-toxic properties, and attractive applications. This article reviews the preparation methods, functional modifications, and widespread applications of IRMOF-3 in various fields.

Since the discovery of IRMOF-3, from the initial solvothermal synthesis method to the emergence and gradual maturity of numerous methods, it has laid a solid foundation for the in-depth research of IRMOF-3. However, there are slight differences in the morphology and properties of IRMOF-3 prepared by different preparation methods, which also poses certain interference and obstacles to the comparative research of IRMOF-3 by different teams.

Functionalized modification has its advantages in designing special functional materials; with the continuous improvement of IRMOF-3 modification methods and the continuous updates of functionalized modification materials, more potential for IRMOF-3 is gradually being developed. Of course, there are also issues such as the low conversion rate of IRMOF-3 modification and complex product characterization methods, especially since its yield or conversion rate has seldom been reported or investigated. Therefore, the yield of functionalization should be enhanced and checked to have better performance and promote the practical development of modified IRMOF-3.

IRMOF-3 and its derivatives are widely used in chemical catalysis, hydrogen storage, material adsorption and separation, carrier materials, fluorescence detection, and other fields. Their derivatives exhibit more prominent characteristics, which also demonstrates the broad application prospects of IRMOF-3 derivatives. Among them, IRMOF-3 is widely used as a carrier material in the loading of drugs and fuels. However, the research on IRMOF-3 as a carrier material in the field of food packaging is only in its infancy. We believe that through unremitting efforts, IRMOF-3 will have great development in the fields of food active packaging and intelligent packaging.

As described in this article, IRMOF-3 has made significant progress in preparation methods, functional modifications, and applications. However, convenient and universal synthesis strategies, rational functionalization modification results, and green and economic applications of IRMOF-3 and its derivatives in more fields are the future development directions of IRMOF-3.

## References

[1] Rocio-Bautista P., Taima-Mancera I., Pasan J., Pino V. (2019). Metal-Organic Frameworks in Green Analytical Chemistry. Separations.

[2] Yu J., Mu C., Yan B.Y., Qin X.Y., Shen C., Xue H.G., Pang H. (2017). Nanoparticle/MOF composites: Preparations and applications. Mater. Horiz..

[3] Liang J.W., Huang Z.Y., Wang K.Y., Zhang L.R., Wan Y.H., Yang T., Zeng H. (2023). Ultrasensitive visual detection of the food-borne pathogen via MOF encapsulated enzyme. Talanta.

[4] Li C.B., Ji Y., Wang Y.P., Liu C.X., Chen Z.Y., Tang J.L., Hong Y.W., Li X., Zheng T.T., Jiang Q. (2023). Applications of Metal-Organic Frameworks and Their Derivatives in Electrochemical CO_2_ Reduction. Nano-Micro Lett..

[5] Gurmessa B.K., Taddesse A.M., Teju E. (2023). UiO-66 (Zr-MOF): Synthesis, Characterization, and Application for the Removal of Malathion and 2, 4-D from Aqueous Solution. Environ. Pollut. Bioavailab..

[6] Begum S., Haikal R.R., Ibrahim A.H., Safy M.A.E., Tsotsalas M., Alkordi M.H. (2020). Flash synthesis for conformal monolithic coatings of the Zr-based metal-organic framework (UiO-66-NH_2_) on non-modified surfaces: Applications in thin-film electrode systems. Surf. Interfaces.

[7] ElHussein E.A.A., Sahin S., Bayazit S.S. (2020). Removal of carbamazepine using UiO-66 and UiO-66/graphene nanoplatelet composite. J. Environ. Chem. Eng..

[8] Liu N., Hu B., Tang K., Xia T., Li F., Quan G., Zhang X., Tang L. (2023). Assembling UiO-66 into layered HTiNbO_5_ nanosheets for efficient photocatalytic CO_2_ reduction. Surf. Interfaces.

[9] Gomar M., Yeganegi S. (2019). Adsorption of 5-Fluorouracil and Thioguanine drugs into ZIF-1, ZIF-3 and ZIF-6 by simulation methods. Mater. Sci. Eng. C-Mater. Biol. Appl..

[10] Kim E., Umar A., Ameen S., Kumar R., Ibrahim A.A., Alhamami M.A.M., Akhtar M.S., Baskoutas S. (2023). Synthesis and characterizations of ZIF-8/GO and ZIF-8/rGO composites for highly sensitive detection of Cu^2+^ ions. Surf. Interfaces.

[11] Nazir M.A., Najam T., Shahzad K., Wattoo M.A., Hussain T., Tufail M.K., Shah S.S.A., Rehman A.U. (2022). Heterointerface engineering of water stable ZIF-8@ZIF-67: Adsorption of rhodamine B from water. Surf. Interfaces.

[12] Alrefaee S.H., Al-bonayan A.M., Alsharief H.H., Aljohani M., Alshammari K.F., Saad F.A., Abumelha H.M., El-Metwaly N.M. (2023). Efficient removal of carbofuran by sono-photo active CdS@MIL based Ti Framework. Surf. Interfaces.

[13] Grad O., Blanita G., Lazar M.D., Mihet M. (2021). Methanation of CO_2_ Using MIL-53-Based Catalysts: Ni/MIL-53-Al_2_O_3_ versus Ni/MIL-53. Catalysts.

[14] Jiang S., Zhao Z., Chen J., Yang Y., Ding C., Yang Y., Wang Y., Liu N., Wang L., Zhang X. (2022). Recent research progress and challenges of MIL-88(Fe) from synthesis to advanced oxidation process. Surf. Interfaces.

[15] Emam H.E., El-Shahat M., Taha M., Abdelhameed R.M. (2022). Microwave assisted post-synthetic modification of IRMOF-3 and MIL-68-NH_2_ onto cotton for Fuel purification with computational explanation. Surf. Interfaces.

[16] Yin D.G., Hu X.L., Cai M.R., Wang K.X., Peng H.Y., Bai J., Xv Y., Fu T.T., Dong X., Ni J. (2022). Preparation, Characterization, and In Vitro Release of Curcumin-Loaded IRMOF-10 Nanoparticles and Investigation of Their Pro-Apoptotic Effects on Human Hepatoma HepG_2_ Cells. Molecules.

[17] Yuksel N., Kose A., Fellah M.F. (2022). A DFT investigation of hydrogen adsorption and storage properties of Mg decorated IRMOF-16 structure. Colloids Surf. A Physicochem. Eng. Asp..

[18] Li H., Eddaoudi M., O’Keeffe M., Yaghi O.M. (1999). Design and synthesis of an exceptionally stable and highly porous metal-organic framework. Nature.

[19] Wang K.X., Cai M.R., Yin D.G., Hu X.L., Peng H.Y., Zhu R.Y., Liu M.T., Xu Y.C., Qu C.H., Ni J. (2022). IRMOF-8-encapsulated curcumin as a biocompatible, sustained-release nano-preparation. Appl. Organomet. Chem..

[20] Yuan S., Feng L., Wang K.C., Pang J.D., Bosch M., Lollar C., Sun Y.J., Qin J.S., Yang X.Y., Zhang P. (2018). Stable Metal-Organic Frameworks: Design, Synthesis, and Applications. Adv. Mater..

[21] Taghavi R., Rostamnia S. (2022). Four-Component Synthesis of Polyhydroquinolines via Unsymmetrical Hantzsch Reaction Employing Cu-IRMOF-3 as a Robust Heterogeneous Catalyst. Chem. Methodol..

[22] Rostamnia S., Morsali A. (2014). Basic isoreticular nanoporous metal-organic framework for Biginelli and Hantzsch coupling: IRMOF-3 as a green and recoverable heterogeneous catalyst in solvent-free conditions. RSC Adv..

[23] Rostamnia S., Morsali A. (2014). Size-controlled crystalline basic nanoporous coordination polymers of Zn_4_O(H_2_N-TA)(3): Catalytically study of IRMOF-3 as a suitable and green catalyst for selective synthesis of tetrahydro-chromenes. Inorganica Chim. Acta.

[24] Hoskins B.F., Robson R. (1990). Design and construction of a new class of scaffolding-like materials comprising infinite polymeric frameworks of 3D-linked molecular rods. A reappraisal of the Zn(CN)_2_ and Cd(CN)_2_ structures and the synthesis and structure of the diamond-related frameworks [N(CH_3_)_4_][CuIZnII(CN)_4_] and CuI[4,4′,4″,4-tetracyanotetraphenylmethane]BF4.xC6H5NO2. J. Am. Chem. Soc..

[25] Wang Z.Q., Cohen S.M. (2007). Postsynthetic covalent modification of a neutral metal-organic framework. J. Am. Chem. Soc..

[26] Wang Z.Q., Cohen S.M. (2009). Postsynthetic modification of metal-organic frameworks. Chem. Soc. Rev..

[27] Zhang F., Chen C., Xu L., Zhang N. (2013). Thermal Decomposition Investigation of Zn_4_O(NH_2_-BDC)(3) (IRMOF-3) and NH_2_-BDC by in situ DRIFTS. Asian J. Chem..

[28] Wu S.H., Ma X., Ran J.Y., Zhang Y.F., Qin F.X., Liu Y. (2015). Application of basic isoreticular nanoporous metal-organic framework: IRMOF-3 as a suitable and efficient catalyst for the synthesis of chalcone. RSC Adv..

[29] Galaco A., Jesus L.T., Freire R.O., de Oliveira M., Serra O.A. (2020). Experimental and Theoretical Studies of Glyphosate Detection in Water by an Europium Luminescent Complex and Effective Adsorption by HKUST-1 and IRMOF-3. J. Agric. Food Chem..

[30] Liu H., Ding W., Lei S.H., Tian X.P., Zhou F.B. (2019). Selective Adsorption of CH_4_/N_−2_ on Ni-based MOF/SBA-15 Composite Materials. Nanomaterials.

[31] Guo H.X., Zheng Z.S., Zhang Y.H., Lin H.B., Xu Q.B. (2017). Highly selective detection of Pb^2+^ by a nanoscale Ni-based metal-organic framework fabricated through one-pot hydrothermal reaction. Sens. Actuators B Chem..

[32] Mai Z.H., Liu D.X. (2019). Synthesis and Applications of Isoreticular Metal-Organic Frameworks IRMOFs-n (n=1, 3, 6, 8). Cryst. Growth Des..

[33] Rathod V.N., Bansode N.D., Thombre P.B., Lande M.K. (2021). Efficient one-pot synthesis of polyhydroquinoline derivatives through the Hantzsch condensation using IRMOF-3 as heterogeneous and reusable catalyst. J. Chin. Chem. Soc..

[34] Isobe T., Arai Y., Yanagida S., Matsushita S., Nakajima A. (2017). Solvothermal preparation and gas permeability of an IRMOF-3 membrane. Microporous Mesoporous Mater..

[35] Su J., Guan J., Fang L., Xu Z., Xiao Y. (2020). Synthesis and Antibacterial Property of IRMOF-3 Grafted with Citral. J. South China Univ. Technol. Nat. Sci. Ed..

[36] Hwang Y.K., Chang J.S., Park S.E., Kim D.S., Kwon Y.U., Jhung S.H., Hwang J.S., Park M.S. (2005). Microwave fabrication of MFI zeolite crystals with a fibrous morphology and their applications. Angew. Chem.-Int. Ed..

[37] Nowicka A., Zielinski M., Debowski M. (2020). Microwave support of the alcoholic fermentation process of cyanobacteria Arthrospira platensis. Environ. Sci. Pollut. Res..

[38] Lee Y.R., Cho S.M., Ahn W.S., Lee C.H., Lee K.H., Cho W.S. (2015). Facile synthesis of an IRMOF-3 membrane on porous Al_2_O_3_ substrate via a sonochemical route. Microporous Mesoporous Mater..

[39] Quan X.Y., Sui S.Y., Huang G.l., Sun L.X., Ma J.J., Wang Y.N. (2022). Synthesis of Metal Organic Frames and Their Application in Food Packaging. Packag. Eng..

[40] Kim H.J., Lee S.B., Choi A.J., Oh J.M. (2019). Zingiber officinale Extract (ZOE) Incorporated with Layered Double Hydroxide Hybrid through Reconstruction to Preserve Antioxidant Activity of ZOE against Ultrasound and Microwave Irradiation. Nanomaterials.

[41] Stock N., Biswas S. (2012). Synthesis of Metal-Organic Frameworks (MOFs): Routes to Various MOF Topologies, Morphologies, and Composites. Chem. Rev..

[42] Razavi S.A.A., Masoomi M.Y., Morsali A. (2018). Morphology-dependent sensing performance of dihydro-tetrazine functionalized MOF toward Al(III). Ultrason. Sonochem..

[43] Whitesides G.M. (2006). The origins and the future of microfluidics. Nature.

[44] Dummann G., Quittmann U., Groschel L., Agar D.W., Worz O., Morgenschweis K. (2003). The capillary-microreactor: A new reactor concept for the intensification of heat and mass transfer in liquid-liquid reactions. Catal. Today.

[45] Mora M.F., Greer F., Stockton A.M., Bryant S., Willis P.A. (2011). Toward Total Automation of Microfluidics for Extraterrestial In Situ Analysis. Anal. Chem..

[46] Jia M.M., Feng Y., Qiu J.H., Yao J.F. (2018). Advances in the synthesis and functionalization of UiO-66 and its applications in membrane separation. Chem. Ind. Eng. Prog..

[47] Faustini M., Kim J., Jeong G.Y., Kim J.Y., Moon H.R., Ahn W.S., Kim D.P. (2013). Microfluidic Approach toward Continuous and Ultrafast Synthesis of Metal-Organic Framework Crystals and Hetero Structures in Confined Microdroplets. J. Am. Chem. Soc..

[48] Mueller U., Schubert M., Teich F., Puetter H., Schierle-Arndt K., Pastre J. (2006). Metal-organic frameworks—Prospective industrial applications. J. Mater. Chem..

[49] Li M.Y., Dinca M. (2015). On the Mechanism of MOF-5 Formation under Cathodic Bias. Chem. Mater..

[50] Wei J.Z., Wang X.L., Sun X.J., Hou Y., Zhang X., Yang D.D., Dong H., Zhang F.M. (2018). Rapid and Large-Scale Synthesis of IRMOF-3 by Electrochemistry Method with Enhanced Fluorescence Detection Performance for TNP. Inorg. Chem..

[51] Huang Z.Y., Liang X.J., Pan X., Zhang S., Zeng T.Y., Chen L.Y., Huang X.W. (2021). Research progress on synthesis and performance optimization of isoreticular metal-organic frameworks( IRMOFs). Ind. Catal..

[52] Zhu M.H., Wu X.M., Niu B.T., Guo H.X., Zhang Y. (2018). Fluorescence sensing of 2,4,6-trinitrophenol based on hierarchical IRMOF-3 nanosheets fabricated through a simple one-pot reaction. Appl. Organomet. Chem..

[53] Ning H.Y., Lu L.X., Xu J., Lu L.J., Pan L., Lin Z.D. (2022). Development of sodium alginate-based antioxidant and antibacterial bioactive films added with IRMOF-3/Carvacrol. Carbohydr. Polym..

[54] Lu C.S., Liu J., Gan L.L., Yang X.M. (2019). Employing Cryptococcus-directed carbon dots for differentiating and detecting m-benzenediol and p-benzenediol. Sens. Actuators B Chem..

[55] Abdelhameed R.M., Carlos L.D., Silva A.M.S., Rocha J. (2013). Near-infrared emitters based on post-synthetic modified Ln^3+^-IRMOF-3. Chem. Commun..

[56] Liu L.L., Tai X.S., Zhou X.J., Xin C.L., Yan Y.M. (2017). Anchorage of Au^3+^ into Modified Isoreticular Metal-Organic Framework-3 as a Heterogeneous Catalyst for the Synthesis of Propargylamines. Sci. Rep..

[57] Tanabe K.K., Wang Z.Q., Cohen S.M. (2008). Systematic functionalization of a metal-organic framework via a postsynthetic modification approach. J. Am. Chem. Soc..

[58] Garibay S.J., Wang Z.Q., Tanabe K.K., Cohen S.M. (2009). Postsynthetic Modification: A Versatile Approach toward Multifunctional Metal-Organic Frameworks. Inorg. Chem..

[59] Custelcean R., Sellin V., Moyer B.A. (2007). Sulfate separation by selective crystallization of a urea-functionalized metal-organic framework. Chem. Commun..

[60] Custelcean R., Moyer B.A., Hay B.P. (2005). A coordinatively saturated sulfate encapsulated in a metal-organic framework functionalized with urea hydrogen-bonding groups. Chem. Commun..

[61] Custelcean R., Moyer B.A., Bryantsev V.S., Hay B.P. (2006). Anion coordination in metal-organic frameworks functionalized with urea hydrogen-bonding groups. Cryst. Growth Des..

[62] Custelcean R., Moyer B.A. (2007). Anion separation with metal-organic frameworks. Eur. J. Inorg. Chem..

[63] Custelcean R., Haverlock T.J., Moyer B.A. (2006). Anion separation by selective crystallization of metal-organic frameworks. Inorg. Chem..

[64] Doyle A.G., Jacobsen E.N. (2007). Small-molecule H-bond donors in asymmetric catalysis. Chem. Rev..

[65] Dugan E., Wang Z.Q., Okamura M., Medina A., Cohen S.M. (2008). Covalent modification of a metal-organic framework with isocyanates: Probing substrate scope and reactivity. Chem. Commun..

[66] Yoo Y., Jeong H.-K. (2012). Generation of covalently functionalized hierarchical IRMOF-3 by post-synthetic modification. Chem. Eng. J..

[67] Wang Z.Q., Cohen S.M. (2008). Tandem modification of metal-organic frameworks by a postsynthetic approach. Angew. Chem.-Int. Ed..

[68] Hamzah H.A., Gee W.J., Raithby P.R., Teat S.J., Mahon M.F., Burrows A.D. (2018). Post-Synthetic Mannich Chemistry on Metal-Organic Frameworks: System-Specific Reactivity and Functionality-Triggered Dissolution. Chem. Eur. J..

[69] Nabipour H., Wang X., Song L., Hu Y. (2021). Organic-inorganic hybridization of isoreticular metal-organic framework-3 with melamine for efficiently reducing the fire risk of epoxy resin. Compos. Part B Eng..

[70] Liu B., Jie S.Y., Bu Z.Y., Li B.G. (2014). A MOF-supported chromium catalyst for ethylene polymerization through post-synthetic modification. J. Mol. Catal. A Chem..

[71] Abdelhameed R.M., El-Naggar M., Taha M., Nabil S., Youssef M.A., Awwad N.S., El Sayed M.T. (2020). Designing a sensitive luminescent probe for organophosphorus insecticides detection based on post-synthetic modification of IRMOF-3. J. Mol. Struct..

[72] Abdelhameed R.M., Carlos L.D., Silva A.M.S., Rocha J. (2015). Engineering lanthanide-optical centres in IRMOF-3 by post-synthetic modification. New J. Chem..

[73] Abdelhameed R.M., Carlos L.D., Rabu P., Santos S.M., Silva A.M.S., Rocha J. (2014). Designing Near-Infrared and Visible Light Emitters by Postsynthetic Modification of Ln^+3^-IRMOF-3. Eur. J. Inorg. Chem..

[74] Liu B., Jie S.Y., Bu Z.Y., Li B.G. (2014). Postsynthetic modification of mixed-linker metal-organic frameworks for ethylene oligomerization. RSC Adv..

[75] Wang Z.Q., Tanabe K.K., Cohen S.M. (2009). Accessing Postsynthetic Modification in a Series of Metal-Organic Frameworks and the Influence of Framework Topology on Reactivity. Inorg. Chem..

[76] Wu Y.J., Zhao S.L., Zhao K., Tu T.X., Zheng J.Z., Chen J., Zhou H.F., Chen D.J., Li S.X. (2016). Porous Fe-N-x/C hybrid derived from bi-metal organic frameworks as high efficient electrocatalyst for oxygen reduction reaction. J. Power Sources.

[77] Ma X., Zhou X.Y., Gong Y., Han N., Liu H.D., Chen Y.F. (2017). MOF-derived hierarchical ZnO/ZnFe_2_O_4_ hollow cubes for enhanced acetone gas-sensing performance. RSC Adv..

[78] Rao Z., Feng K., Tang B.B., Wu P.Y. (2017). Surface Decoration of Amino-Functionalized Metal Organic Framework/Graphene Oxide Composite onto Polydopamine-Coated Membrane Substrate for Highly Efficient Heavy Metal Removal. ACS Appl. Mater. Interfaces.

[79] Borousan F., Yousefi F., Ghaedi M. (2019). Removal of Malachite Green Dye Using IRMOF-3 MWCNT-OH-Pd-NPs as a Novel Adsorbent: Kinetic, Isotherm, and Thermodynamic Studies. J. Chem. Eng. Data.

[80] Sohrabnezhad S., Moghadamy S. (2022). Zinc oxide nanorods incorporated magnetic isoreticular metal-organic framework for photodegradation of dyes. J. Mol. Struct..

[81] Li D., Dai X.P., Zhang X., Zhuo H.Y., Jiang Y., Yu Y.B., Zhang P.F., Huang X.L., Wang H. (2017). Silver nanoparticles encapsulated by metal-organic-framework give the highest turnover frequencies of 10(5) h(−1) for three component reaction by microwave-assisted heating. J. Catal..

[82] Gao G., Di J.Q., Zhang H.Y., Mo L.P., Zhang Z.H. (2020). A magnetic metal organic framework material as a highly efficient and recyclable catalyst for synthesis of cyclohexenone derivatives. J. Catal..

[83] Vu T.V., Kosslick H., Schulz A., Harloff J., Paetzold E., Radnik J., Kragl U., Fulda G., Janiak C., Tuyen N.D. (2013). Hydroformylation of olefins over rhodium supported metal-organic framework catalysts of different structure. Microporous Mesoporous Mater..

[84] Gascon J., Aktay U., Hernandez-Alonso M.D., van Klink G.P.M., Kapteijn F. (2009). Amino-based metal-organic frameworks as stable, highly active basic catalysts. J. Catal..

[85] Cortese R., Duca D. (2011). A DFT study of IRMOF-3 catalysed Knoevenagel condensation. Phys. Chem. Chem. Phys..

[86] Xamena F., Cirujano F.G., Corma A. (2012). An unexpected bifunctional acid base catalysis in IRMOF-3 for Knoevenagel condensation reactions. Microporous Mesoporous Mater..

[87] Dai T.L., Zhang Y.M., Chu G., Zhang J. (2017). Preparation and Characterization of IRMOF-3 and Their Catalytic Performances in Knoevenagel Condensation. New Chem. Mater..

[88] Zhou X., Zhang Y., Yang X.G., Zhao L.Z., Wang G.Y. (2012). Functionalized IRMOF-3 as efficient heterogeneous catalyst for the synthesis of cyclic carbonates. J. Mol. Catal. A Chem..

[89] Nuri A., Vucetic N., Smatt J.H., Mansoori Y., Mikkola J.P., Murzin D.Y. (2019). Pd Supported IRMOF-3: Heterogeneous, Efficient and Reusable Catalyst for Heck Reaction. Catal. Lett..

[90] Cheng J., Mao Y.X., Guo H., Qian L., Shao Y., Yang W.J., Park J.Y. (2022). Synergistic and efficient catalysis over Bronsted acidic ionic liquid BSO_3_HMIm HSO_4_-modified metal-organic framework (IRMOF-3) for microalgal biodiesel production. Fuel.

[91] Zhang Y.M., Zhang J., Li X.F., Chu G., Tian M.M., Qun C.S. (2016). Preparation of Fe_3_O_4_@IRMOF-3/Pd and Its Catalytic Performance as a Heterogeneous Multifunctional Catalyst. Chem. J. Chin. Univ..

[92] Zhang J.N., Yang X.H., Guo W.J., Wang B., Zhang Z.H. (2017). Magnetic Metal-Organic Framework CoFe_2_O_4_@SiO_2_@IRMOF-3 as an Efficient Catalyst for One-Pot Synthesis of Functionalized Dihydro-2-oxopyrroles. Synlett.

[93] Li W.G., Li G., Liu D. (2016). Synthesis and application of core-shell magnetic metal-organic framework composites Fe_3_O_4_/IRMOF-3. RSC Adv..

[94] Yao S., Wang Y.T., Chi J.J., Yu Y.R., Zhao Y.J., Luo Y., Wang Y.G. (2022). Porous MOF Microneedle Array Patch with Photothermal Responsive Nitric Oxide Delivery for Wound Healing. Adv. Sci..

[95] Rosi N.L., Eckert J., Eddaoudi M., Vodak D.T., Kim J., O’Keeffe M., Yaghi O.M. (2003). Hydrogen storage in microporous metal-organic frameworks. Science.

[96] Rowsell J.L.C., Yaghi O.M. (2006). Effects of functionalization, catenation, and variation of the metal oxide and organic linking units on the low-pressure hydrogen adsorption properties of metal-organic frameworks. J. Am. Chem. Soc..

[97] Rowsell J.L.C., Millward A.R., Park K.S., Yaghi O.M. (2004). Hydrogen sorption in functionalized metal-organic frameworks. J. Am. Chem. Soc..

[98] Wang Z.Q., Tanabe K.K., Cohen S.M. (2010). Tuning Hydrogen Sorption Properties of Metal-Organic Frameworks by Postsynthetic Covalent Modification. Chem. Eur. J..

[99] Barea E., Montoro C., Navarro J.A.R. (2014). Toxic gas removal—Metal-organic frameworks for the capture and degradation of toxic gases and vapours. Chem. Soc. Rev..

[100] Tian F.M., Zhang X.H., Chen Y.L. (2016). Amino-functionalized metal-organic framework for adsorption and separation of dichloromethane and trichloromethane. RSC Adv..

[101] Britt D., Tranchemontagne D., Yaghi O.M. (2008). Metal-organic frameworks with high capacity and selectivity for harmful gases. Proc. Natl. Acad. Sci. USA.

[102] Wang X.L., Fan H.L., Tian Z., He E.Y., Li Y., Ju S.G. (2014). Adsorptive removal of sulfur compounds using IRMOF-3 at ambient temperature. Appl. Surf. Sci..

[103] Li D., Wang H., Zhang X., Sun H., Dai X.P., Yang Y., Ran L., Li X.S., Ma X.Y., Gao D.W. (2014). Morphology Design of IRMOF-3 Crystal by Coordination Modulation. Cryst. Growth Des..

[104] Altintas C., Avci G., Daglar H., Azar A.N.V., Velioglu S., Erucar I., Keskin S. (2018). Database for CO_2_ Separation Performances of MOFs Based on Computational Materials Screening. ACS Appl. Mater. Interfaces.

[105] Farrusseng D., Daniel C., Gaudillere C., Ravon U., Schuurman Y., Mirodatos C., Dubbeldam D., Frost H., Snurr R.Q. (2009). Heats of Adsorption for Seven Gases in Three Metal-Organic Frameworks: Systematic Comparison of Experiment and Simulation. Langmuir.

[106] Karra J.R., Walton K.S. (2010). Molecular Simulations and Experimental Studies of CO_2_, CO, and N_−2_ Adsorption in Metal-Organic Frameworks. J. Phys. Chem. C.

[107] Ding S.M., Dong Q.L., Hu J.W., Xiao W.M., Liu X.H., Liao L.Q., Zhang N. (2016). Enhanced selective adsorption of CO_2_ on nitrogen-doped porous carbon monoliths derived from IRMOF-3. Chem. Commun..

[108] Ullah S., Bustam M.A., Assiri M.A., Al-Sehemi A.G., Kareem F.A.A., Mukhtar A., Ayoub M., Gonfa G. (2019). Synthesis and characterization of iso-reticular metal-organic Framework-3 (IRMOF-3) for CO_2_/CH_4_ adsorption: Impact of post-synthetic aminomethyl propanol (AMP) functionalization. J. Nat. Gas Sci. Eng..

[109] Zhang X., Wang N.N., Li H.B., Wang Z., Wang H.T. (2023). IRMOF-3 nanosheet-filled glass fiber membranes for efficient separation of hydrogen and carbon dioxide. Sep. Purif. Technol..

[110] Gu Z.Y., Jiang J.Q., Yan X.P. (2011). Fabrication of Isoreticular Metal-Organic Framework Coated Capillary Columns for High-Resolution Gas Chromatographic Separation of Persistent Organic Pollutants. Anal. Chem..

[111] Wang H., Zhao X.Y., Xu J.W., Shang Y.Z., Wang H., Wang P., He X.T., Tan J. (2021). Determination of quinolones in environmental water and fish by magnetic metal organic frameworks based magnetic solid-phase extraction followed by high-performance liquid chromatography-tandem mass spectrometry. J. Chromatogr. A.

[112] Kalashgrani M.Y., Babapoor A., Mousavi S.M., Feizpoor S., Hashemi S.A., Binazadeh M., Chiang W.H., Lai C.W. (2023). Synthesis of Isoreticular Metal Organic Framework-3 (IRMOF-3) Porous Nanostructure and Its Effect on Naphthalene Adsorption: Optimized by Response Surface Methodology. Separations.

[113] Ma Y., Wang L.L., Feng R.J., Wang J.L., Cai T.Z. (2021). Preparation of anion exchange membrane by incorporating IRMOF-3 in quaternized chitosan. Polym. Bull..

[114] Cai M.R., Qin L.Y., Pang L.N., Ma B.R., Bai J., Liu J., Dong X.X., Yin X.B., Ni J. (2020). Amino-functionalized Zn metal organic frameworks as antitumor drug curcumin carriers. New J. Chem..

[115] Yang B.C. (2012). Preparation of Anti-Tumor Drug 5-FU Loaded Folate-Conjugated Porous Metal-Organic Frameworks. Master’s Thesis.

[116] Li L. (2015). RGD Peptide Modified Preparation and Drug Loading Norcantharidin Nanoparticles of Metal Organic Framework IRMOF-3 Pharmacokinetic Study. Master’s Thesis.

[117] Xu T. (2015). Active Target Study on RGD Peptide Mediated Nano Metal Organic Framework IRMOF-3 Loading Cantharidin. Ph.D. Thesis.

[118] Zhang Y.H. (2015). The Preparation and Targeting Property Preliminary Research on RGD Modified Nano Metal Organic Frameworks IRMOF-3. Master’s Thesis.

[119] Sahu S.K., Maiti S., Pramanik A., Ghosh S.K., Pramanik P. (2012). Controlling the thickness of polymeric shell on magnetic nanoparticles loaded with doxorubicin for targeted delivery and MRI contrast agent. Carbohydr. Polym..

[120] Chowdhuri A.R., Singh T., Ghosh S.K., Sahu S.K. (2016). Carbon Dots Embedded Magnetic Nanoparticles @Chitosan @Metal Organic Framework as a Nanoprobe for pH Sensitive Targeted Anticancer Drug Delivery. ACS Appl. Mater. Interfaces.

[121] Chowdhuri A.R., Bhattacharya D., Sahu S.K. (2016). Magnetic nanoscale metal organic frameworks for potential targeted anticancer drug delivery, imaging and as an MRI contrast agent. Dalton Trans..

[122] Sanaei-Rad S., Ghasemzadeh M.A., Aghaei S.S. (2022). Synthesis and structure elucidation of ZnFe_2_O_4_/IRMOF-3/GO for the drug delivery of tetracycline and evaluation of their antibacterial activities. J. Organomet. Chem..

[123] Mozaffari F., Razavian S.M.H., Ghasemzadeh M.A. (2023). Encapsulation of Allopurinol in GO/CuFe_2_O_4_/IR MOF-3 Nanocomposite and In Vivo Evaluation of Its Efficiency. J. Pharm. Innov..

[124] Li X.Y., Guan Q.X., Shang Y.Z., Wang Y.H., Lv S.W., Yang Z.X., Wang R., Feng Y.F., Li W.N., Li Y.J. (2021). Metal-organic framework IRMOFs coated with a temperature-sensitive gel delivering norcantharidin to treat liver cancer. World J. Gastroenterol..

[125] Wu Y.P., Luo Y.G., Zhou B., Mei L., Wang Q., Zhang B. (2019). Porous metal-organic framework (MOF) Carrier for incorporation of volatile antimicrobial essential oil. Food Control.

[126] He C.C., Yu H.H., Sun J., Zhou C., Li X., Su Z.M., Liu F.B., Khakhinov V. (2022). Luminescent composites by in-suit encapsulating dye in IRMOF-3 for ratiometric temperature sensing and tunable white light emission. Dye. Pigment..

[127] Liu X.M., Xu Y.H., Jiang D.L. (2012). Conjugated Microporous Polymers as Molecular Sensing Devices: Microporous Architecture Enables Rapid Response and Enhances Sensitivity in Fluorescence-On and Fluorescence-Off Sensing. J. Am. Chem. Soc..

[128] Wang M., Guo L., Cao D.P. (2018). Metal-organic framework as luminescence turn-on sensor for selective detection of metal ions: Absorbance caused enhancement mechanism. Sens. Actuators B Chem..

[129] Wang S.H., Li P.F., Wang Z.R., Zhang N. (2013). Postsynthetic Covalent Modification of IRMOF-3 and Its Luminescence Properties. J. Instrum. Anal..

[130] Wang M., Guo L., Cao D.P. (2018). Amino-Functionalized Luminescent Metal-Organic Framework Test Paper for Rapid and Selective Sensing of SO_2_ Gas and Its Derivatives by Luminescence Turn-On Effect. Anal. Chem..

[131] Senthil R.A., Selvi A., Arunachalam P., Amudha L.S., Madhavan J., Al-Mayouf A.M. (2017). A sensitive electrochemical detection of hydroquinone using newly synthesized alpha-Fe_2_O_3_-graphene oxide nanocomposite as an electrode material. J. Mater. Sci.-Mater. Electron..

[132] Nsanzamahoro S., Zhang Y., Wang W.F., Ding Y.Z., Shi Y.P., Yang J.L. (2021). Fluorescence “turn-on” of silicon-containing nanoparticles for the determination of resorcinol. Microchim. Acta.

[133] Cao Q., Yu Q.Y., Li Z., Huang Z.Z., Jia Q. (2022). Rhodamine B functionalized luminescent metal-organic frameworks for ratiometric fluorescence sensing of hydroquinone. J. Mater. Chem. B.

[134] Devi S., Shaswat S., Kumar V., Sachdev A., Gopinath P., Tyagi S. (2020). Nitrogen-doped carbon quantum dots conjugated isoreticular metal-organic framework-3 particles based luminescent probe for selective sensing of trinitrotoluene explosive. Microchim. Acta.

[135] Bhardwaj N., Bhardwaj S.K., Mehta J., Nayak M.K., Deep A. (2016). Bacteriophage conjugated IRMOF-3 as a novel opto-sensor for S. arlettae. New J. Chem..

[136] Cui Y.J., Zhu F.L., Chen B.L., Qian G.D. (2015). Metal-organic frameworks for luminescence thermometry. Chem. Commun..

